# Thrombocyte-derived Dickkopf1 promotes macrophage polarization in the Bleomycin-induced lung injury model

**DOI:** 10.3389/fimmu.2023.1247330

**Published:** 2023-12-15

**Authors:** Eun-Ah Sung, Min Hee Park, SuJeong Song, Hasan Alanya, Octavian Henegariu, Jinze Liu, E Zeynep Erson-Omay, Patricia J. Sime, Wook-Jin Chae

**Affiliations:** ^1^Department of Microbiology and Immunology, Virginia Commonwealth University School of Medicine, Richmond, VA, United States; ^2^Massey Comprehensive Cancer Center, Virginia Commonwealth University, Richmond, VA, United States; ^3^Department of Neurosurgery, Yale University School of Medicine, New Haven, CT, United States; ^4^Department of Biostatistics, Virginia Commonwealth University School of Medicine, Richmond, VA, United States; ^5^Department of Internal Medicine, Virginia Commonwealth University School of Medicine, Richmond, VA, United States; ^6^Phillips Oral Health Research Institute, Virginia Commonwealth University School of Dentistry, Richmond, VA, United States

**Keywords:** Dickkopf1, macrophage, polarization, thrombocyte, inflammation

## Abstract

Immune responses are crucial to maintaining tissue homeostasis upon tissue injury. Upon various types of challenges, macrophages play a central role in regulating inflammation and tissue repair processes. While an immunomodulatory role of Wnt antagonist Dickkopf1 (DKK1) has been implicated, the role of Wnt antagonist DKK1 in regulating macrophage polarization in inflammation and the tissue repair process remains elusive. Here we found that DKK1 induces gene expression profiles to promote inflammation and tissue repair in macrophages. Importantly, DKK1 induced various genes, including inflammation and tissue repair, via JNK (c-jun N-terminal kinase) in macrophages. Furthermore, DKK1 potentiated IL-13-mediated macrophage polarization and activation. The co-inhibition of JNK and STAT6 markedly decreased gene expressions *relevant to* inflammation and fibrosis by DKK1 and IL-13. Interestingly, thrombocyte-specific deletion of DKK1 in mice reduced collagen deposition and decreased Arg1, CD206, HIF1α, and IL1β protein expressions in monocyte-derived alveolar macrophages in the acute sterile bleomycin (BLM)-induced lung injury model. These data suggested that thrombocytes communicate with macrophages via DKK1 to orchestrate inflammation and repair in this model. Taken together, our study demonstrates DKK1’s role as an important regulatory ligand for macrophage polarization in the injury-induced inflammation and repair process in the lung.

## Introduction

Upon various environmental challenges, including infectious pathogens, allergens, and chemicals, tissue injuries cause inflammatory immune responses. Immune responses are vital biological processes to remove the origin of inflammation and maintain tissue homeostasis (1). Tissue repair and regeneration following tissue injury are augmented to reinstate tissue homeostasis. Dysregulation of these processes leads to chronic inflammation or organ fibrosis.

The Wnt signaling pathway is an essential regulator of tissue homeostasis since it promotes cell differentiation, proliferation, immune cell function, and tissue repair (2–4). Dysregulation of Wnt signaling pathways and aberrant expression of Wnt antagonists have been reported in various diseases such as asthma, autoimmunity, and cancer (5, 6). Multiple studies have shown that elevated protein levels of Wnt antagonist Dickkopf1 (DKK1) were associated with chronic inflammatory diseases (7–9). DKK1’s function as a pro-inflammatory immunomodulator in type 2 and type 17 inflammatory disease models has been highlighted (10, 11). While DKK1 induces type 2 inflammation via CD4^+^ T cells in allergen-induced asthma and a parasitic infection model, whether DKK1 regulates other immune cells that play a central role in inflammation and tissue repair remains elusive.

Among immune cells, macrophages drive the initial immune responses following injury by producing chemokines and inflammatory mediators (12). Macrophages are an important source of numerous soluble mediators, chemokines, and growth factors that recruit and stimulate various cell types involved in inflammation and tissue repair (13). These features make macrophages a key immune cell subset to regulate immune responses and tissue repair.

Upon injury, monocytes are recruited into the wound site from the bone marrow and are differentiated into macrophages. These recruited macrophages play a vital role in wound healing by temporarily and mechanistically driving inflammation and tissue repair processes (14–17). Perturbation of phenotypic and functional changes in these macrophages results in miscommunications with other cells involved in wound healing, leading to chronic inflammatory diseases (13).

Type 2 inflammation is featured by increased type 2 cytokines such as IL-13 and type 2 inflammation-associated macrophage (18). The macrophage polarization by type 2 cytokines promotes tissue repair (19). IL-13 promotes the recruitment and activation of macrophages in type 2 cell-mediated inflammation, inducing Arginase-1 (Arg1), CD206, Fra-2, and Fizz1 (19–22). Matrix metalloproteinases (MMPs) are enzyme involved in the maintenance and remodeling of extracellular matrix (ECM), and tissue inhibitor of metalloproteinase 1 (TIMP1) plays a regulatory role as inhibitors of MMPs (23, 24). Along with type 2 cell-mediated inflammation, a reduction of MMP expression or increased TIMP1 expression in macrophages promotes ECM protein deposition in the injured lung (25). Aberrant activation of macrophages dysregulates homeostatic crosstalk with other cells for tissue repair, leading to pathological fibrosis (21, 26, 27).

In this study, we addressed the role of DKK1 in regulating the polarization of macrophages. We sought to delineate signaling pathways for DKK1-mediated macrophage polarization using pharmacological and genetic approaches. We identified thrombocyte-derived DKK1 as a critical regulator of macrophage polarization using thrombocyte-specific deletion of Dkk1 in mice upon BLM-induced lung injury.

## Materials and methods

### Mice

C57Bl/6J mice (#000664), STAT6 knockout (KO) (#005977), and Pf4-cre mice (#008535) were purchased from the Jackson Laboratory and have been bred in our mouse facility. Dkk1 flox mice sperm was purchased from the EMMA mouse repository (EMMA: 09872) and rederived by Virginia Commonwealth University (VCU) transgenic core facility. All mouse protocols were approved by the VCU Animal Care and Use Committee (IACUC) approval in accordance with the Association for Assessment and Accreditation of Laboratory Animal Care International (AAALAC).

### Bone marrow-derived macrophage differentiation and activation

To isolate bone marrow (BM) cells, femurs and tibias were obtained from 8- to 10-week-old C57Bl/6J or STAT6 KO mice. The BM cells were collected and suspended in RBC lysis buffer (420302, BioLegend). For BMDM differentiation, the BM cells were gently resuspended to BMDM differentiation medium (DMEM supplemented with 20% FBS (100-106, Gemini Bio Products), 1% Pen/Strep, 1% Sodium pyruvate, 1% GlutaMax, 1% MEM-NEAA and 20 ng/ml of murine M-CSF (576404, BioLegend)) as described previously (28–31). Six days after seeding the BM cells, the cells were cultured in BMDM cultivation medium (RPMI 1640 supplemented with 10% FBS, 1% Pen/Strep, 1% Sodium pyruvate, 1% GlutaMax, 1% MEM-NEAA and 5 ng/ml of murine M-CSF). For macrophage activation, the BMDMs were treated with 100 ng/ml of LPS (L6529, Sigma-Aldrich) or 2 ng/ml of recombinant murine IL-13 (575902, BioLegend) with or without 30 ng/ml of recombinant mouse DKK1 (759602, BioLegend) for 48 hours.

For inhibitors, JNK inhibitor (2 μM, SP600125, A4604, APExBIO), p38 MAPK inhibitor (5 μM, SB203580, 1202, Tocris), SGK-1 inhibitor (2 μM, GSK 653094, 3572, Tocris), mTOR inhibitor (100 pM, Rapamycin, S1039, Selleckchem), GSK-3β inhibitor (100 nM, TWS110, B1540, APExBIO), ERK inhibitor (1 μM, PD98059, A1663, APExBIO), and PI3K inhibitor (1 μM, LY 294002, A8250, APExBIO) were treated with cytokines for 48 hours where indicated. The antibodies used for flow cytometry are listed in **Supplementary Table 1**. Data were collected on a FACSCanto (BD Biosciences) and analyzed using FlowJo software (Tree Star).

### RNA extraction and quantitative RT-PCR

Total RNA from BMDMs was extracted according to the manufacturer’s instructions using TRIzol reagent (15596026, Thermo Fisher Scientific). RNA purification with the isolated RNA was performed using RNeasy Plus Mini Kit (74136, QIAGEN), according to the manufacturer’s instructions. Reverse transcription of total RNA was performed using RNA to cDNA EcoDry Premix kit (639548, Takara Bio). qPCR was performed using PowerUp SYBR Green Master Mix (A25742, Thermo Fisher Scientific). The reaction was detected on a QuantStudio 3 or 5 Flex Real-Time PCR (Thermo Fisher Scientific). The mRNA levels of target genes were normalized by comparing them to the mRNA level of TATA-box binding protein (Tbp) control using the 2^-ΔΔCt^ method (32). Relative mRNA levels were quantified by setting the Tbp control as 1000 for normalization. Primers used for qPCR are listed in **Supplementary Table 2**.

### Bulk RNA seq

Total RNA from BMDMs was extracted using TRIzol, and purified RNA was isolated using the Rneasy Plus Mini Kit reagent according to the manufacturer’s instructions. The quality of RNA-seq reads was assessed with FastQC v0.11.9. The reads were aligned using STAR aligner v2.7.6a to reference genome GRCm39 (33). Raw gene counts of mapped reads were aggregated using featureCounts (34). Following the acquisition of the count matrix, an unbiased analysis of differential gene expression (DEG) was performed with Bioconductor package DESeq2 v1.30.0 using the normalized and filtered counts per gene from the RNA-seq analysis (35). DEGs with adjusted p-value < 0.05 and an absolute log2-fold change > 1 were considered statistically significant. Functional Enrichment Analysis was conducted using enrichGO from the clusterProfiler package (36, 37). The DEGs are listed in **Supplementary Data Sheet 1** (Control vs. DKK1), **2** (Control vs. IL-13), **3** (Control vs. IL-13 **+** DKK1), and **4** (IL-13 vs IL-13 + DKK1). Raw data were deposited to the GEO repository.

### Hemavet analysis

Peripheral blood (75 μl) was collected from Dkk1^fl/fl^ and Pf4-cre Dkk1 ^fl/fl^ mice using the retro-orbital sinus sampling method. The Hemavet 950FS (Drew Scientific) was used to count immune cells, platelets, and red blood cells.

### Enzyme-linked immunosorbent assay

Concentrations of circulating DKK1 in plasma samples were measured by ELISA according to the manufacturer’s protocol (MKK100, R&D systems).

### Bleomycin-induced lung injury model

Bleomycin Sulfate (BLM; S121415, Selleckchem) (4 U/kg) was dissolved in 40 μl of PBS for each mouse per oropharyngeal challenge. 8- to 10-week-old C57Bl/6J mice, Pf4-cre Dkk1^fl/fl^ mice, and their littermate controls were challenged with BLM on day 0. Mice were sacrificed, and lungs were harvested and perfused on day 14. For platelet depletion, anti-mouse CD41 antibody (100 μg, clone: MWReg30, 133940, BioLegend) and isotype Rat IgG (100 μg, 400457, BioLegned) were intraperitoneally injected on day 0 upon BLM challenge.

For flow cytometry analysis, single-cell lung homogenates were prepared by collagenase digestion (280 U/ml Collagenase type IV (LS004188, Worthington Biochemicals), 2% (v/v) FBS (100-106, Gemini Bio Products), and 40 μg/ml DNase I (10104159001, Roche) in PBS using the gentleMACS Octo Dissociator (Miltenyi Biotec). After blocking the Fc Receptor, cells were stained with Zombie Aqua Fixable Viability Kit (423102, BioLegend). Subsequently, the cells were stained with fluorescent-conjugated antibodies against different cell surface antigens. Following surface staining, cells were fixed with IC fixation buffer (00-8222-49, Thermo Fisher Scientific) for intracellular staining. The antibodies used are listed in **Supplementary Table 1**. FACSCanto (BD Biosciences) was used for flow cytometry, and data were analyzed by FlowJo software (Tree Star).

### Immunohistochemistry, immunofluorescence staining, and western blot

Lungs were fixed with 10% formalin (23-245685, Fisher Scientific) embedded in paraffin. Lung tissue sections (5 μm thick) were used. For H&E staining, the nuclei were stained by Hematoxylin (MHS32, Sigma-Aldrich), and the cytoplasm was stained by Eosin (HT110132, Sigma-Aldrich). For immunohistochemistry (IHC) and immunofluorescence staining (IF), sodium citrate buffer (10 mM, pH 6.0) was used for antigen retrieval. Endogenous peroxidases were blocked using 3% H_2_O_2_. The sections were incubated with 5% BSA and subsequently stained with primary antibody (Goat polyclonal anti-DKK1, AF1096, R&D systems (6 μg/ml); Rabbit polyclonal anti-CD41 PA5-22307, Thermo Fisher Scientific (1:50)). For IHC, the sections were stained with 5 μg/ml of HRP-conjugated secondary antibody (HRP-conjugated mouse anti-goat IgG, sc-2354, Santa Cruz Biotechnology). Sections were treated with the ABC reagent (PK-7100, Vector Laboratories) and developed using a DAB substrate kit (SK-4100, Vector Laboratories). The nuclei were stained by Hematoxylin. The sections were mounted with the mounting solution (H-5000, Vector Laboratories), and images were acquired using Vectra Polaris (Akoya Biosciences).

For IF, the sections were stained with secondary antibodies (Alexa Fluor 488-conjugated donkey anti-goat IgG, 705-545-147, Jackson ImmunoResearch; Alexa Fluor 568-conjugated goat anti-rabbit IgG, A-11036, Invitrogen). Sections were incubated with a Vector TrueVIEW Autofluorescence Quenching Kit (SP-8500, Vector Laboratories) and mounted in VECTASHIELD Vibrance Antifade Mounting Medium with DAPI. Images were acquired using an EVOS FL Auto (Thermo Fisher Scientific) or All-in-one Keyence fluorescence microscope (BZ-X810).

For Western blot, lung homogenate lysates were separated by SDS-PAGE and transferred onto a polyvinylidene difluoride membrane. The membrane was blocked and immunoblotted with primary antibodies against DKK1 (AF1096, R&D systems) at 1:1500 dilution and β-actin (3700, Cell Signaling) at 1:5000 dilution. After incubation with HRP-conjugated secondary antibodies, the membrane was developed using a WesternBright™ Sirius kit (K-12043-D10, Advansta). Bands were detected and quantified using the Chemidoc and ImageJ and normalized to β-actin protein levels.

### Masson’s Trichrome staining and quantification of collagen deposition

After deparaffinization, the lung tissue sections (5 μm thick) were fixed in Bouin’s Solution (11750-32, IHC world). They were stained with Masson Trichrome Stain Kit (IW-3006, IHC world) according to the manufacturer’s protocol and mounted with the mounting solution (H-5000, Vector Laboratories). Images were acquired using Vectra Polaris (Akoya Biosciences). Images were saved as TIFF using Phenochart, a slider viewer software (Akoya Biosciences). The “Color Deconvolution” plugin (Masson Trichrome) in Fiji software was used for image processing. Images were deconvoluted in their color components. The green component of Masson Trichrome was measured using the “Threshold” tool. The Threshold value of the deconvoluted images was adjusted, and the same threshold value was applied to all processed images. The selected area of the image was analyzed using the “Measure” tool. Total collagen deposition was quantified from the entire tissue section. Pictures from ten fields were randomly taken in the parenchyma region of the lungs, excluding big bronchiole and arterial structures, for quantification of parenchyma collagen deposition.

### Statistical analysis

Statistically significant differences were analyzed by Student’s t-test, one-way ANOVA analysis, and two-way ANOVA analysis with Bonferroni’s *post hoc* test with GraphPad Prism software (version 9.4.1, GraphPad Software Inc.). Data are represented as means ± SD. P values < 0.05 were considered significant.

## Results

### DKK1 induces various gene expression profiles including inflammation and tissue repair in macrophages

**Figure 1:**
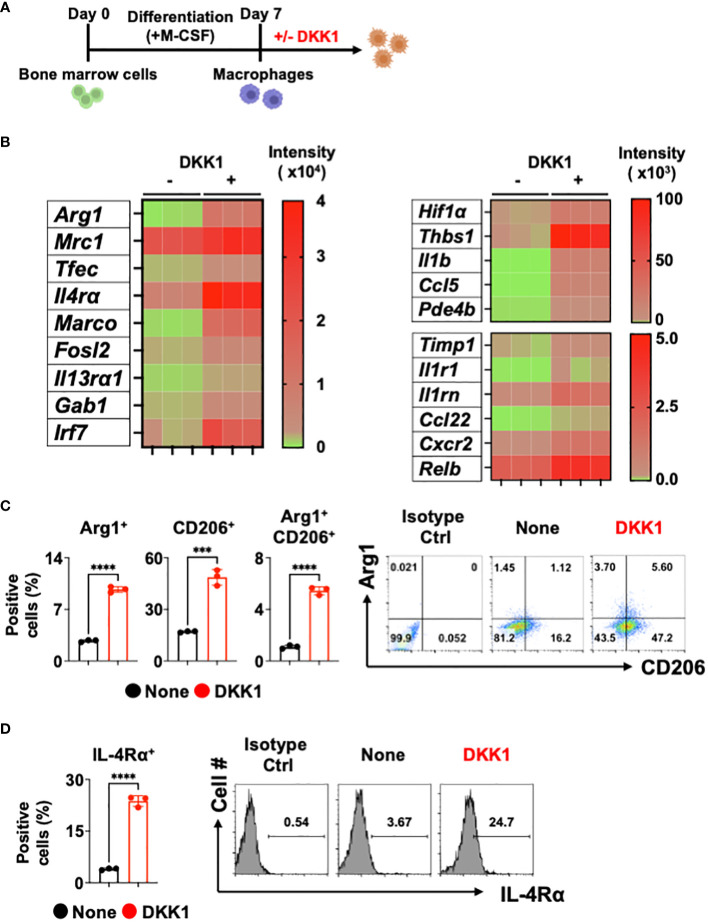
DKK1 induces *various gene expressions including* inflammation and fibrosis in macrophages. **(A-D)** Experimental scheme of bone marrow-derived macrophages (BMDMs) created with Biorender.com **(A)**. BMDMs were treated with DKK1 for 24 hours prior to bulk RNA sequencing (RNAseq) **(B)** and for 48 hours prior to flow cytometry analysis **(C, D)**. A representative of three independent experiments is shown **(C, D)**. Student’s t-test was performed for **(C, D)**. ****p < 0.0001, ***p < 0.001. See also **Supplementary Figure 1**.

It has been shown that DKK1 is an immune modulator in multiple inflammatory disease models (10, 11, 38). Whether DKK1 can modulate macrophages was not investigated. Given that type 2 cytokine-activated macrophages are important for promoting type 2 inflammation and tissue repair, we used bone marrow-derived macrophages (BMDMs) to explore DKK1-mediated gene expressions *in vitro* (**Figure 1A** and **Supplementary Figure 1A**). Interestingly, bulk RNA seq analyses for differentially expressed genes (DEGs) and heatmap analyses showed that DKK1 upregulated a variety of genes, including ones that are involved in type 2 cytokine-mediated macrophage activation, tissue repair, and fibrosis (**Figure 1B** and **Supplementary Figure 1B****).** Transcription factor EC (Tfec) transcribes IL-4 receptor α (IL-4Rα) expression to promote IL-4-activated macrophage polarization (39). The scavenger receptor, macrophage receptor with collagenous structure (MARCO), polarized macrophages to a type 2 cytokine-induced phenotype in asbestos-induced fibrosis (40). Irf7 mRNA expression was upregulated by DKK1 (**Figure 1B**). Interferon regulatory factor 7 (IRF7) controls the pro- to anti-inflammatory macrophage phenotype switch in response to chronic exposure to transforming growth factor β1 (TGFβ1) (41). Further analyses using Gene Ontology (GO) terms revealed that DKK1-treated macrophages exhibited enrichment in essential components of the immune responses, including leukocyte migration, positive regulation of response to external stimuli, cytokine-mediated signaling pathways, and positive regulation of cell adhesion (**Supplementary Figure 1C**).

We further examined these points with DEG profile and heatmap analyses and found that DKK1 induces a variety of genes known to play key roles in inflammation and tissue repair in macrophages (**Figure 1B** and **Supplementary Figure 1B**). Hypoxia plays an important role in pulmonary fibrosis pathogenesis by increasing pro-resolving genes in the BLM-induced lung injury model (42, 43). A recent study has shown that the Wnt/β-catenin pathway promoted an inflammatory activity of macrophages following viral infection via enhanced Hif1α expression (44). We found that the MMP inhibitor Timp1 gene expression was increased by DKK1, suggesting that macrophage-mediated degradation of collagens may be decreased (**Figure 1B**). Thbs1 is a major activator of TGFβ1, and inhibition of Thbs1 decreases collagen deposition in the BLM model (45, 46). A notable increase in Thbs1 expression by DKK1 was detected (**Figure 1B**). DKK1 increased mRNA expressions of Il1b, its receptor Il1r1, and interleukin 1 receptor antagonist Il1rn. They have been reported to be crucial in developing pulmonary inflammation and fibrosis (47–50). Pro-resolving chemokines and chemokine receptor gene expressions were elevated by DKK1 (**Figure 1B**). It has been shown that Ccl22 and Cxcr2 promote the pathogenesis of pulmonary fibrosis (51, 52). Ccl5 is a chemokine that recruits eosinophils and promotes allergic airway inflammation (53). A recent study reported that Arg1^+^ lung macrophages expressed greater Ccl5 when compared to Arg1^-^ macrophages (54). The inhibition of phosphodiesterase 4 (PDE4) reduced lung fibrosis in the BLM-induced lung injury model (55). mRNA expression of Pde4b was increased by DKK1 (**Figure 1B**). It has been recently shown that inhibition of nuclear factor-kappa B (NF-κB) protects the lung against BLM-induced injury in mice (56). DKK1 increased the mRNA expression of Relb, a member of the NF-κB family (**Figure 1B**). We confirmed these findings using qPCR analyses and showed examples (**Supplementary Figure 1D**).

Since DKK1 induced several genes known to be induced by IL-13, we examined the protein expression levels of Arg1, CD206, and IL-4Rα. DKK1 significantly increased Arg1^+^ and CD206^+^ cell populations (**Figure 1C**). IL-4Rα is shared by two type 2 cytokines, IL-13 and IL-4, to promote tissue repair in macrophages (57). IL-4Rα expression was increased by DKK1, indicating that DKK1 increases sensitivity to type 2 cytokines (**Figure 1D**). Further analyses showed that IL-4Rα expressions were increased by DKK1 in Arg1^+^ and CD206^+^ cells (**Supplementary Figure 1E**). Collectively, our results indicated that DKK1 induces distinct gene expression profiles, including inflammation and tissue repair without type 2 cytokines.

### STAT6 is not a key mediator of DKK1-mediated macrophage polarization

**Figure 2:**
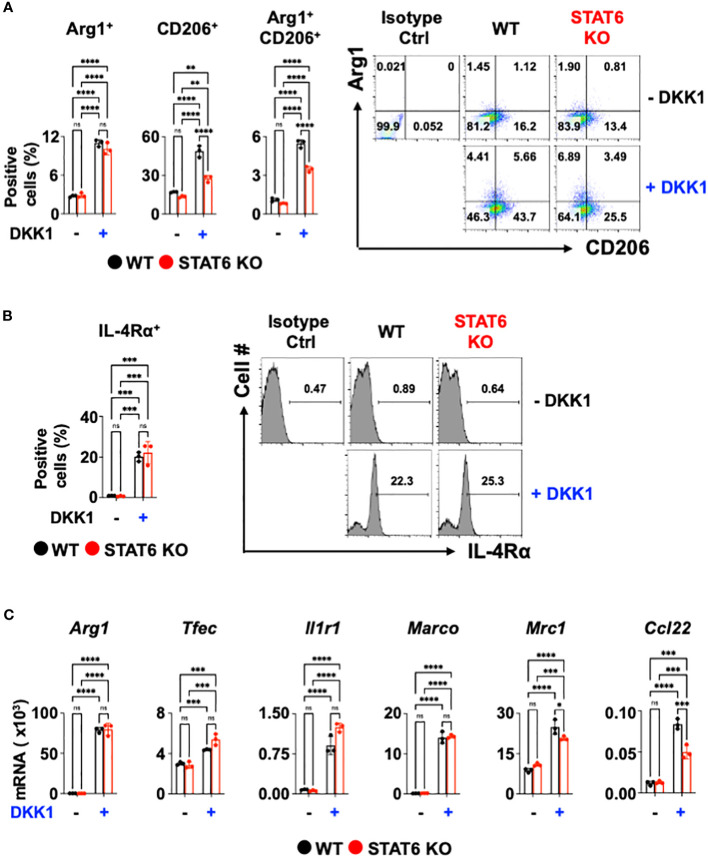
STAT6 is not a critical mediator of DKK1-mediated macrophage polarization. **(A-C)** BMDMs from C57Bl/6J mice (WT) or STAT6 knockout mice (STAT6 KO) were treated with or without DKK1 for 48 hours prior to flow cytometry analysis **(A, B)** and for 24 hours prior to qPCR **(C)**. All experiments are representative of three independent experiments. A two-way ANOVA analysis with Bonferroni’s *post hoc* test was performed for **(A-C)**. ****p < 0.0001, ***p < 0.001, **p < 0.005, *p < 0.05, ns, not significant. See also **Supplementary Figure 2**.

Next, we investigated the DKK1-mediated macrophage gene expression signaling pathways. The signal transducer and activator of transcription 6 (STAT6) is an important transcription factor for type 2 cytokine-activated macrophage polarization (58). To test whether DKK1 induces macrophage polarization markers via STAT6, STAT6-deficient BMDMs were used. Interestingly, STAT6-deficiency did not decrease the DKK1-mediated Arg1^+^ cell population increase (**Figure 2A**). STAT6-deficiency only partially decreased CD206^+^ and Arg1^+^CD206^+^ cell populations (**Figure 2A**). IL-4Rα^+^ cell populations increased by DKK1 were unaffected by STAT6 deficiency (**Figure 2B**).

We questioned whether DKK1 induces other type 2 cytokine-activated macrophage-related genes or pro-resolving gene expressions via STAT6. Consistent with **Figure 2A**, Arg1, Tfec, Il1r1, and Marco mRNA expressions induced by DKK1 were unaffected by STAT6-deficiency (**Figure 2C**). STAT6 deficiency did not markedly decrease Mrc1, and Ccl22 mRNA expressions increased by DKK1, but the effect was statistically significant (**Figure 2C**). STAT6 deficiency largely decreased type 2 cytokine-activated macrophage marker gene expression by IL-13 (**Supplementary Figure 2**). Our data suggest that DKK1 utilizes differential signaling pathways from IL-13.

### JNK is a primary mediator of DKK1-mediated macrophage polarization

**Figure 3:**
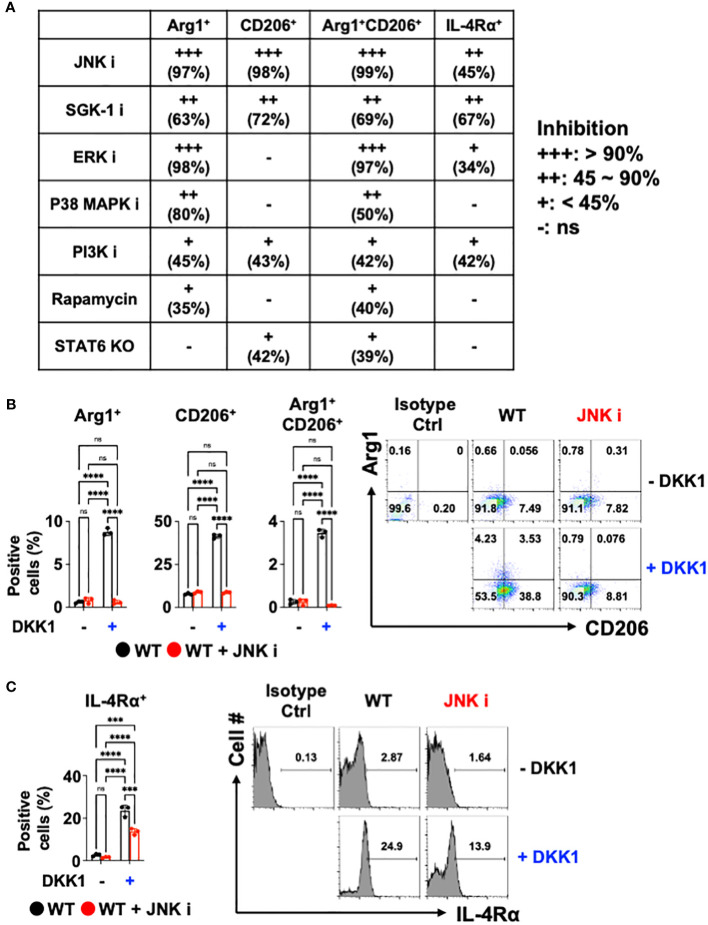
JNK is a primary mediator of DKK1-mediated macrophage polarization. **(A)** Table summarizing the pharmacological inhibition (ns: no significant effect). **(B, C)** BMDMs from C57Bl/6J mice (WT) were incubated with JNK inhibitor with or without DKK1 for 48 hours prior to flow cytometry analysis. All experiments are representative of three independent experiments. A two-way ANOVA analysis with Bonferroni’s *post hoc* test was performed for **(A, B)**. ****p < 0.0001, ***p < 0.001, ns, not significant. See also **Supplementary Figure 3**.

Previous reports showed that DKK1 utilizes a variety of signaling pathways. For example, DKK1 activated the c-Jun N-terminal kinase (JNK) signaling pathway to increase tumor cell growth (59–61). Mammalian targets of Rapamycin (mTOR) and phosphoinositide 3-kinase (PI3K) promote type 2 cytokine-activated macrophage polarization and the development of pulmonary fibrosis (62–66). ERK pathway contributes to type 2 cytokine-activated macrophage polarization in breast cancer (67). DKK1 induced T helper 2 (Th2) cells by p38 MAPK or serum glucocorticoid kinase-1 (SGK-1) pathway (10). We decided to test whether these pathways are utilized to mediate macrophage polarization by DKK1. Pharmacological approaches were used to test whether DKK1 utilizes these multiple pathways to induce macrophage polarization. We summarized the pharmacological inhibition results of each pathway (**Figure 3A**).

SGK-1 and PI3K inhibition regulated these markers significantly but to a lesser extent (**Figure 3A** and **Supplementary Figure 3A**). The inhibition of ERK and p38 MAPK failed to reduce CD206^+^ cell populations and IL-4Rα expressions. mTOR pathways did not show marked change upon DKK1-mediated increase in CD206^+^ and IL-4Rα^+^ cell populations (**Figure 3A** and **Supplementary Figure 3A**). STAT6-mediated pathway inhibition using STAT6 KO BMDMs was compared to the pharmacological inhibition results, indicating that STAT6’s role in macrophage polarization by DKK1 was marginal (**Figure 3A**).

Interestingly, Arg1^+^, CD206^+^, and Arg1^+^CD206^+^ cell populations were notably decreased by JNK inhibition in the presence of DKK1 (**Figure 3B**). JNK inhibition decreased DKK1-mediated IL-4Rα expressions (**Figure 3C**). Further, other genes that were activated by DKK1 were notably decreased by JNK inhibition in the presence of DKK1 (**Supplementary Figure 3B**). Collectively, our results suggest that JNK, but not STAT6 inhibition, served as the primary mediator and was the most effective in decreasing the DKK1-mediated signaling pathways in macrophages.

### IL-13-mediated macrophage polarization is enhanced by DKK1

To test DKK1’s role in classically activated macrophage versus IL-13-activated macrophage polarization conditions, lipopolysaccharide (LPS) or IL-13 were used with or without DKK1. Classically activated macrophages are activated by bacterial components (e.g., LPS) and pro-inflammatory Th1 cytokine IFN-γ (15, 68). IL-13 plays an important role in macrophage polarization and type 2 inflammation-mediated fibrosis (69). Compared to the IL-13 alone-treated group, Arg1, Mrc1, Tfec, Fosl2, and Timp1 were upregulated by co-treatment of DKK1 and IL-13 (**Supplementary Figure 4A**). LPS-induced Nos2 mRNA expression level was substantially decreased by DKK1 (**Supplementary Figure 4B**). DKK1 partially decreased the expression of other LPS-induced classically activated macrophage-related genes (Il-6, Il1b, Nlrp3) but not Tnfα (**Supplementary Figure 4B**).

**Figure 4:**
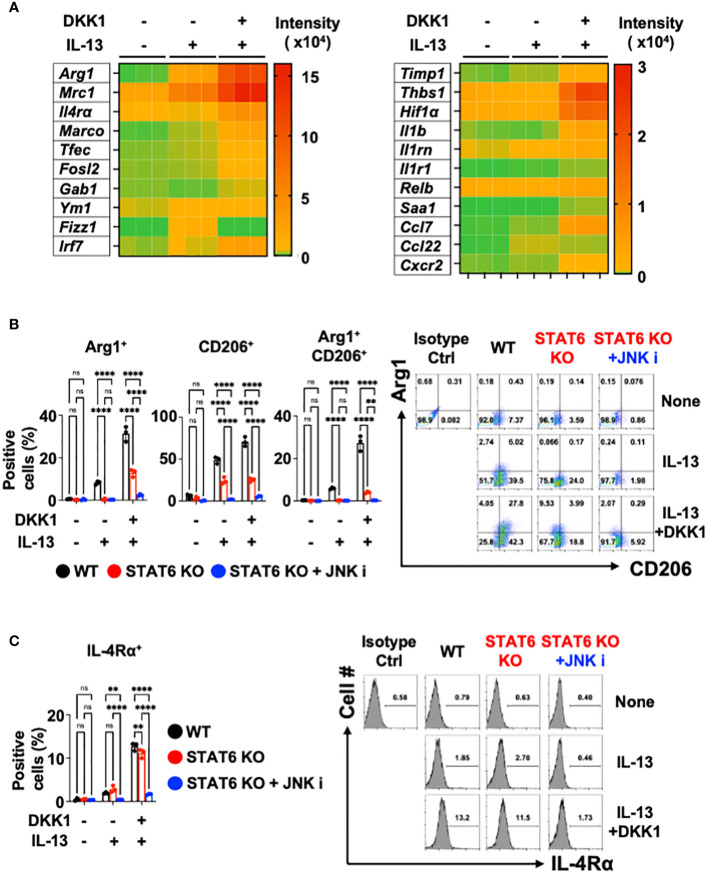
Co-inhibition of STAT6 and JNK blocked DKK1 and IL-13-induced gene expressions in macrophages. **(A)** BMDMs were treated with IL-13 with or without DKK1 for 24 hours prior to bulk RNA sequencing (RNA seq). **(B, C)** BMDMs from C57Bl/6J mice (WT) or STAT6 knockout mice (STAT6 KO) were treated with IL-13 and/or DKK1 either with or without JNK inhibitor for 48 hours. Cell populations were then measured by flow cytometry. A representative of at least two independent experiments is shown **(B, C)**. A two-way ANOVA analysis with Bonferroni’s *post hoc* test was performed for **(B, C)**. ****p < 0.0001, **p < 0.005, ns, not significant. See also **Supplementary Figure 4**.

We further investigated whether DKK1 can enhance IL-13-mediated macrophage polarization. Notably, DKK1 enhanced IL-13-mediated gene expressions in BMDMs, as indicated in the heatmap (**Figure 4A**). Ym1 and Fizz1 mRNA expression levels were increased by IL-13 alone, but no such effect was observed with DKK1. DKK1 enhanced IL-13-mediated gene expression profiles in pulmonary fibrosis (**Figure 4A**). DKK1 and IL-13 co-treatment induced pro-resolving genes such as Timp1, Thbs1, Hif1α, Il1b, and Cxcr2. These genes were increased by co-treatment but not by IL-13 alone, indicating that the co-treatment potentiated DKK1-mediated gene expressions (**Figure 4A**). In line with the heatmap results, the bar plot of DEGs showed that the co-treatment of DKK1 and IL-13 increased the expression of genes, such as Arg1 and Mrc1 in comparison to the group treated with IL-13 alone (**Supplementary Figure 4C**). GO terms analysis showed that the co-treatment group enhanced processes related to positive regulation of cell adhesion, cytokine-mediated signaling pathway, and leukocyte migration compared to the group treated with IL-13 alone (**Supplementary Figure 4D**). In summary, DKK1 enhanced IL-13-mediated macrophage polarization and activation gene expressions.

### DKK1 and IL-13-mediated macrophage gene expressions are abrogated by JNK and STAT6 co-inhibition

Upon DKK1 and IL-13 co-treatment, Arg1^+^, CD206^+^, and Arg1^+^CD206^+^ cell populations were substantially increased compared to IL-13-treated BMDMs (**Figure 4B**). IL-4Rα^+^ expression was increased by DKK1 and IL-13 co-treatment (**Figure 4C**). STAT6 is an important mediator of IL-13-mediated macrophage-related genes such as Arg1, Mrc1, Fizz1, and Ym1 (58, 70). Upon DKK1 and IL-13 co-treatment, Arg1^+^ and CD206^+^ cell populations were decreased by 53% and 47% in STAT6-deficient BMDMs (**Figure 4B**). IL-4Rα^+^ cells were decreased only by 15% in STAT6-deficient BMDMs (**Figure 4C**). These results showed that STAT6 deficiency alone is insufficient to abrogate DKK1 and IL-13-mediated macrophage phenotypes.

Since the JNK inhibitor effectively abrogated DKK1-mediated gene expressions, we questioned whether the co-inhibition of STAT6 and JNK pathways is sufficient to inhibit DKK1 and IL-13-mediated macrophage marker expressions. STAT6 and JNK inhibition significantly decreased most Arg1^+^ and CD206^+^ cell population induction and IL-4Rα^+^ expression (**Figures 4B, C**). In line with these results, the co-inhibition markedly decreased several genes that were induced by DKK1 and IL-13 (**Supplementary Figure 4E**). Taken together, we showed that STAT6 and JNK co-inhibition effectively inhibit DKK1 and IL-13-induced gene expressions for macrophage activation and polarization.

### Thrombocyte-derived DKK1 modulates macrophages in the BLM-induced injury

**Figure 5:**
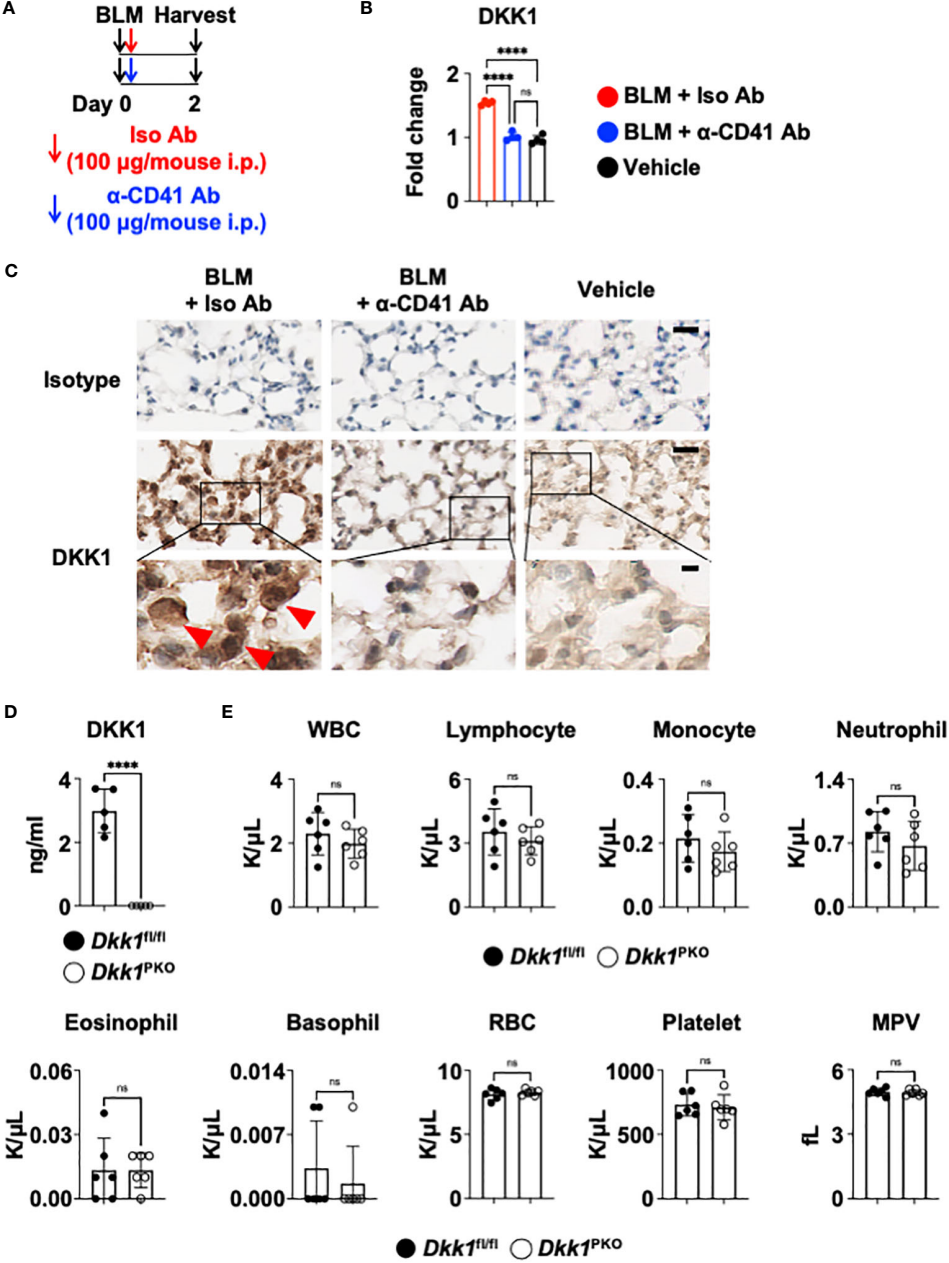
Thrombocyte is a major source of DKK1. **(A-C)** Upon BLM challenge, C57Bl/6J mice (n = 4 per group) were intraperitoneally injected with an isotype control antibody (Iso Ab) or anti-CD41a antibody (α-CD41 Ab) on day 0. The lungs were harvested on day 2 **(A)**. DKK1 protein levels were analyzed by IHC and Image J **(B)**. Representative IHC images are shown **(C)**. The boxed areas are shown in higher magnification below. DKK1-positive cells are indicated by red arrows. Bar = 20 μm (top and middle panels); 5 μm (bottom panels). **(D)** Peripheral blood was collected from Dkk1^PKO^ mice (n = 5) and their Dkk1^fl/fl^ littermate controls (n = 5). Circulating DKK1 protein levels were analyzed by DKK1 ELISA. **(E)** Hemavet analysis of inflammatory cells in the peripheral blood was performed from Dkk1^PKO^ mice (n = 6) and their Dkk1^fl/fl^ littermate controls (n = 6). Shown are white blood cells (WBC), lymphocytes, monocytes, neutrophils, eosinophils, basophils, red blood cells (RBC), platelets, and mean platelet volume (MPV). A one-way ANOVA analysis with Bonferroni’s *post hoc* test was performed for **(B)**. Student’s t-test was performed for **(D, E)**. ****p < 0.0001, ns, not significant. See also **Supplementary Figure 5**.

Recent studies have demonstrated that DKK1 from thrombocytes is a pro-inflammatory immunomodulator in type 2 and type 17 inflammation (10, 11). It has been shown that platelets regulate macrophage functions (71). To examine whether the DKK1 protein level is from thrombocytes, we probed the co-localization of DKK1 and CD41 proteins 14 days after the BLM challenge. Notably, DKK1 was primarily co-localized with the CD41^+^ thrombocytes (**Supplementary Figure 5A**). The lungs from BLM-treated mice showed increased co-localization of DKK1 and CD41. To test whether thrombocyte is a major source of DKK1 in the acute BLM-induced lung injury, we treated anti-CD41 antibody or isotype control antibody intraperitoneally upon BLM challenge (**Figure 5A**). DKK1 protein levels were quantitated in the lung upon BLM challenge using DKK1 immunohistochemistry (IHC) images. DKK1 protein levels in the lung were increased in BLM-treated mice compared to vehicle-treated mice (**Figures 5B, C**). Thrombocyte depletion using anti-CD41 antibody largely inhibited the increase of DKK1 protein levels upon BLM challenge, indicating that thrombocytes are sources of DKK1 upon BLM-induced lung injury (**Figures 5B, C**).

To further determine whether thrombocytes are a primary source of DKK1, we generated thrombocyte-specific DKK1-deficient mice (Pf4-cre Dkk1^fl/fl^ and hereafter Dkk1^PKO^). We confirmed that Dkk1^PKO^ had very little DKK1 in the plasma (**Figure 5D**). Next, we performed hematological analyses of Dkk1^PKO^ mice and their littermate control Dkk1^fl/fl^ mice. We found little difference in hematological parameters between Dkk1^PKO^ mice and Dkk1^fl/fl^ mice blood, indicating that Dkk1^PKO^ mice have no abnormalities to test the role of DKK1 in the BLM model (**Figure 5E**).

**Figure 6:**
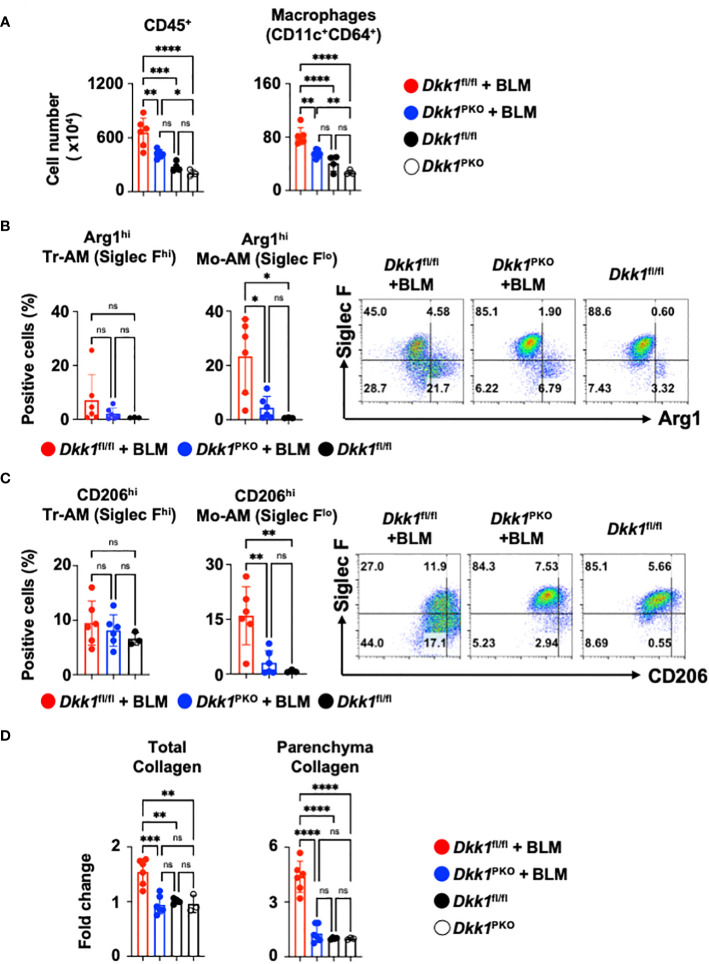
Thrombocyte-derived DKK1 modulates macrophages upon BLM-induced lung injury. **(A-D)** Dkk1^PKO^ mice and their Dkk1^fl/fl^ littermate controls were challenged with BLM. On day 14, after the challenge, the lungs were harvested. **(A)** Total leukocytes (CD45^+^) and macrophages from lung homogenates were quantitated by flow cytometry. **(B, C)** Arg1^hi^ **(B)** and CD206^hi^ **(C)** tissue resident-alveolar macrophages (Tr-AMs) and monocyte-derived alveolar macrophages (Mo-AMs) from lung homogenates were quantified by flow cytometry. **(D)** Total collagen and parenchyma collagen deposition were determined by Masson staining and ImageJ. Dkk1^fl/fl^ + BLM (n = 6); Dkk1^PKO^ + BLM (n = 6); Dkk1^fl/fl^ (n= 3-4); Dkk1^PKO^ (n = 3). A one-way ANOVA analysis with Bonferroni’s *post hoc* test was performed for **(A–D)**. ****p < 0.0001, ***p < 0.001, **p < 0.005, *p < 0.05, ns, not significant. See also **Supplementary Figure 6**.

To investigate the role of thrombocyte-derived DKK1 for lung macrophages upon BLM-induced lung injury, Dkk1^PKO^ mice and their littermate control Dkk1^fl/fl^ mice were challenged with BLM. Dkk1^PKO^ mice and Dkk1^fl/fl^ mice were examined for DKK1 protein levels in the lung upon BLM challenge. BLM-treated Dkk1^PKO^ mice showed minimal DKK1 protein levels in the lung, close to vehicle-treated Dkk1^fl/fl^ and Dkk1^PKO^ mice by DKK1 IHC (**Supplementary Figure 5B**). In line with these data, DKK1 protein levels were substantially reduced in BLM-treated Dkk1^PKO^ mice compared with BLM-treated Dkk1^fl/fl^ mice (**Supplementary Figure 5C**). BLM-treated Dkk1^fl/fl^ mice showed increased CD45^+^ leukocytes and macrophage infiltration in the lung (**Figure 6A**). The increased numbers of CD45^+^ leukocytes and macrophages were significantly reduced in Dkk1^PKO^ mice compared to Dkk1^fl/fl^ mice upon BLM challenge (**Figure 6A** and **Supplementary Figure 6A**). Consistent with these findings, Hematoxylin and eosin (H&E) staining showed that inflammation and leukocyte infiltration were substantially reduced in BLM-treated Dkk1^PKO^ mice compared to BLM-treated Dkk1^fl/fl^ mice (**Supplementary Figure 6B**).

We questioned whether DKK1 regulates type 2 inflammation-related macrophage markers Arg1 and CD206 in the lung macrophages from Dkk1^PKO^ mice upon BLM-induced lung injury. Arg1^+^ and CD206^+^ macrophages were substantially decreased in the lungs of Dkk1^PKO^ mice compared to Dkk1^fl/fl^ mice upon acute BLM injury (**Figures 6B, C**). Monocyte-derived alveolar macrophages (Mo-AMs) were differentiated by their Siglec F expression levels from tissue resident-alveolar macrophages (Tr-AMs) and promoted lung fibrosis (72). We observed that Tr-AM cells showed little change in largely Arg1 expression, while Mo-AMs showed a decrease in Arg1 expression in BLM-treated Dkk1^PKO^ mice (**Figure 6B**). Consistently, Tr-AM showed little difference in CD206 expression, while Mo-AM CD206 expression was decreased in Dkk1^PKO^ mice (**Figure 6C**). Interestingly, we found that CD206^+^ Mo-AMs showed increased levels of IL-4Rα, Hif1α, IL-1β, and Cxcr2 proteins (**Supplementary Figure 6C**). These protein expressions decreased in Dkk1^PKO^ mice compared to those from littermate control mice (**Supplementary Figure 6C**). Lastly, we investigated whether collagen deposition by BLM injury is reduced in Dkk1^PKO^ mice. Upon BLM challenge, Dkk1^PKO^ mice showed reduced collagen deposition in the whole lung area and lung parenchyma compared to Dkk1^fl/fl^ mice (**Figure 6D** and **Supplementary Figure 6D**). Collectively, our data suggested that thrombocyte-derived DKK1 regulates macrophage phenotypes in the BLM-induced lung injury model.

## Discussion

In this study, we showed that DKK1 induces genes that are important for macrophage polarization and activation. Dkk1^PKO^ mice markedly decreased inflammation, macrophage phenotypes, and collagen deposition upon BLM-induced lung injury, suggesting that thrombocyte-derived DKK1 serves as a key immunomodulator for macrophages in this model.

Previously, we demonstrated that DKK1 employed STAT6, a critical mediator, to polarize CD4^+^ T cells into Th2 cells (10). The limited use of STAT6 by DKK1 in macrophages suggests that DKK1-mediated gene expressions are mediated by atypical signaling pathways from other type 2 cytokines. The marked decrease in DKK1-mediated macrophage gene expressions by JNK inhibition demonstrates that DKK1-mediated signaling is differential among immune cell types. Another example of DKK1-mediated gene expressions is that DKK1 did not induce well-known alternatively activated macrophage markers, such as Fizz1. Fizz1-deficient alternatively activated macrophages promoted exacerbated type 2 cell-mediated inflammation, revealing an unrecognized role of macrophage Fizz1 (73). Our results suggest that more studies are warranted regarding the mechanisms and drivers of macrophages for inflammation and tissue repair phenotypes in the lung and other organs using different murine model systems and human specimens.

The induction of Arg1^+^ macrophage induction by IL-13 and DKK1 *in vitro* and the reduction of Arg1^+^ lung macrophages in Dkk1^PKO^ mice *in vivo* suggest the sustained elevation of thrombocyte-derived DKK1 in the lung by environmental insults induce pathological pulmonary inflammation. The importance of IL-9-mediated Arg1^+^ macrophages in allergic asthma has been recently reported (54). It has been reported that IL-9 expressing Th9 cells and IL-9 promotes pulmonary fibrosis in mice, coinciding with monocyte-derived alveolar macrophages (Mo-AMs) expressing Arg1 in the BLM-induced lung fibrosis model (72, 74, 75). It would be worth investigating whether DKK1 and IL-13 or IL-9 are interdependent in macrophage polarization. Further analyses will be required on whether other environmental insults trigger type 2 cell-mediated inflammation by IL-13 or IL-9 without DKK1. We found that DKK1-induced CD206^+^ and CD206^+^/Arg1^+^ macrophages were affected by STAT6 deficiency, while IL-4Rα and other genes remained unaffected. Our bulk RNA seq results further revealed that DKK1’s role in macrophage polarization and activation is not limited to AAM-like gene markers. The use of Arg1-YFP reporter mice bred to STAT6 KO mice and macrophage-specific ablation of Arg1 using LyzM-cre and CD11c-cre is warranted for further investigation on DKK1-induced CD206^+^ and CD206^+^/Arg1^+^ macrophages using multiple tissue injury and repair models including the BLM-induced lung injury model.

A previous study showed that Mo-AMs are the primary pulmonary fibrosis driver in the BLM challenge model, while the Mo-AM gene expression profiles are not exclusively alternatively activated macrophages (72). Our data are in line with this finding that Hif1α or IL-1β protein expressions were regulated by DKK1 in addition to Arg1 and CD206. Our results from differential gene expressions by DKK1 suggest that atypical and differential signaling pathways induced by DKK1 contribute to the heterogeneous macrophage phenotypes contributing to pathological inflammation, suggesting that more studies are warranted on multiple mechanisms in which macrophage promotes lung inflammation and fibrosis. The importance and the role of DKK1 in pathological fibrosis in multiple organs warrant future studies.

Dkk1^PKO^ mice showed no abnormal hematological phenotypes, and no notable differences were observed in leukocyte infiltration at steady state between Dkk1^PKO^ mice and their littermate control mice. Our data indicate that the prolonged expression of DKK1 upon organ or tissue injuries can induce aberrant macrophage polarization and activation that may lead to progressive organ or tissue fibrosis.

It can be postulated that inflammation demands its biological priority for host survival when the cost of tissue damage by DKK1-driven inflammation outweighs the benefit of sustaining tissue homeostasis by Wnt-driven cell proliferation and differentiation. In this regard, the presence of both DKK1 and Wnt upon tissue injury may indicate that injury-induced DKK1 from platelets may override Wnt-mediated cell proliferation and differentiation. Further studies regarding spatiotemporal kinetics and the relative abundance of two ligands in the local injury site will provide more insights for fine-tuning tissue repair processes by DKK1 and Wnt3a in a cell-type-dependent manner.

We showed that thrombocyte-derived DKK1 regulated macrophage polarization upon BLM-induced lung injury using Dkk1^PKO^ mice. Further studies using human platelets from pulmonary fibrosis patients are required to overcome the limitation of our study utilizing the acute BLM-induced lung injury mouse model. A previous study may shed light on this point, as it was demonstrated that macrophage polarization was induced by platelet-rich human plasma (76).

Taken together, our study provides a novel insight into DKK1’s role in the regulatory mechanisms that promote inflammation via macrophage polarization, placing DKK1 as an important immunoregulatory ligand to regulate macrophages in the BLM-induced lung injury model.

## Data availability statement

The data presented in the study are deposited in the NCBI GEO repository, accession number GSE247398.

## Ethics statement

The animal study was approved by Jolene Windle, Virginia Commonwealth University. The study was conducted in accordance with the local legislation and institutional requirements.

## Author contributions

E-AS, MP, and W-JC designed experiments, analyzed data, and wrote the manuscript. E-AS, MP, and SS performed experiments. HA, JL, and EE-O analyzed bioinformatics for RNA sequencing. PS gave input and guidance on the lung injury model. OH provided input for qPCR experiments. W-JC supervised all aspects of the project. All of the authors read and commented on the manuscript.
