# Supplementary material for: Thrombocyte-derived Dickkopf1 promotes macrophage polarization in the Bleomycin-induced lung injury model
Source: Front Immunol. 2023 Dec 15;14:1247330. doi: 10.3389/fimmu.2023.1247330 (PMC10757334; doi:10.3389/fimmu.2023.1247330)

Supplementary Figure 1

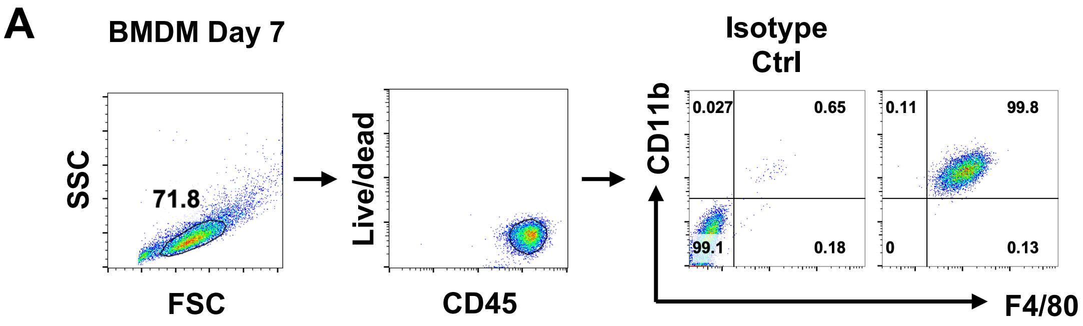

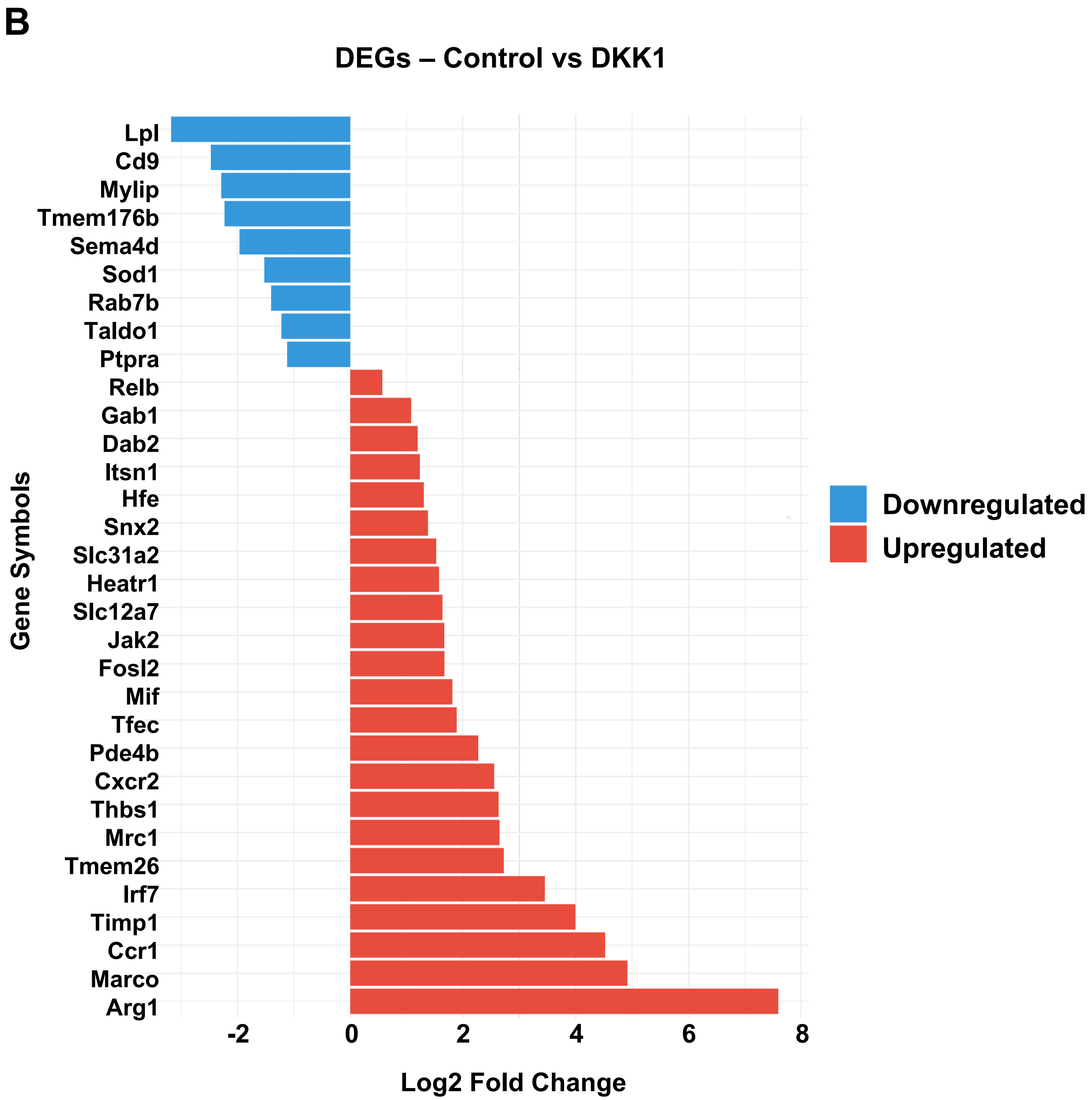

Supplementary Figure 1 (continued)

C

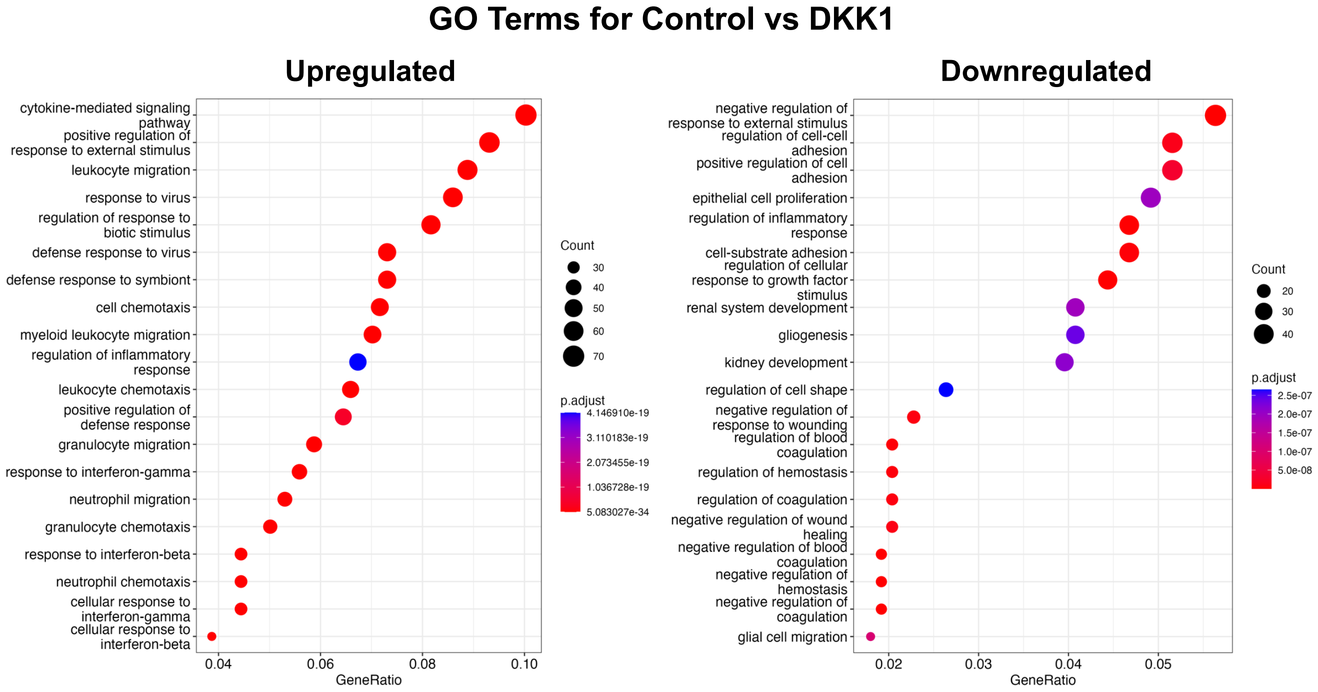

D

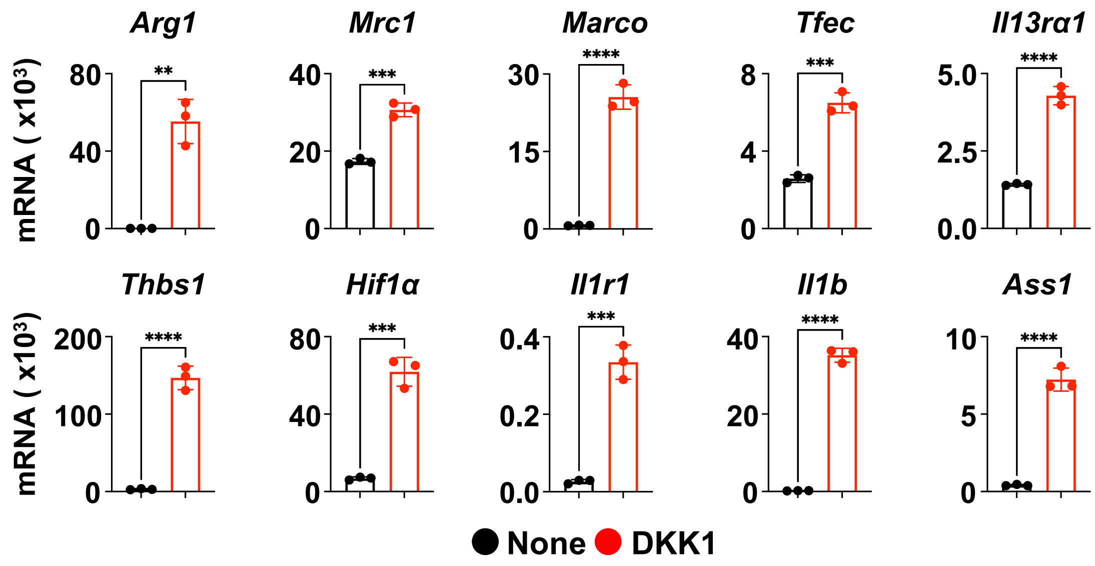

Supplementary Figure 1 (continued)

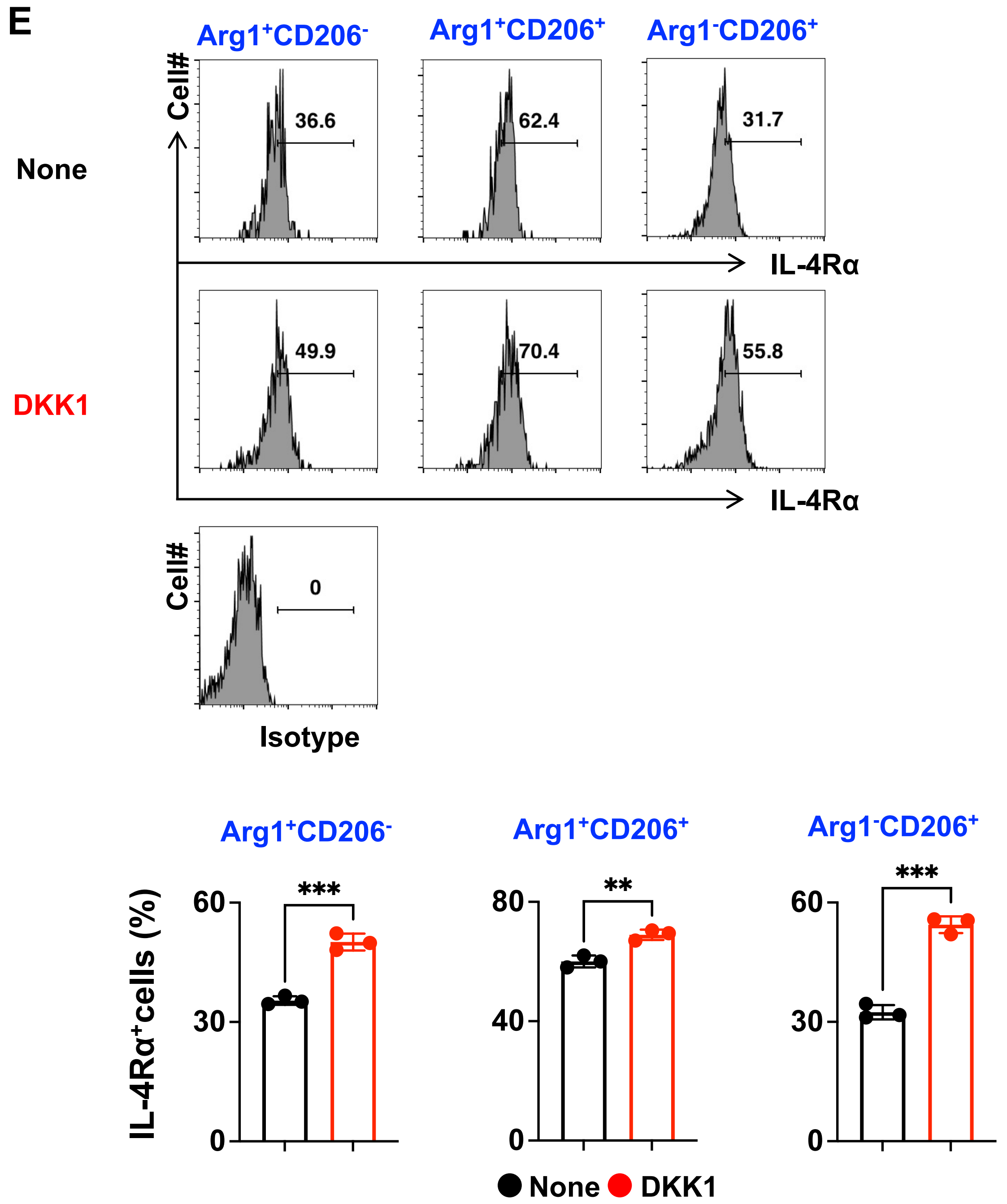

Supplementary Figure 2

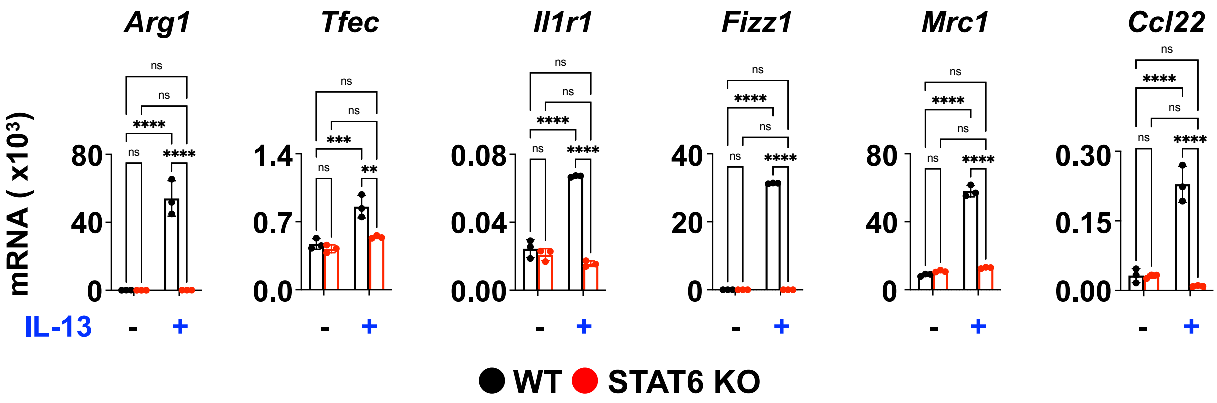

Supplementary Figure 3

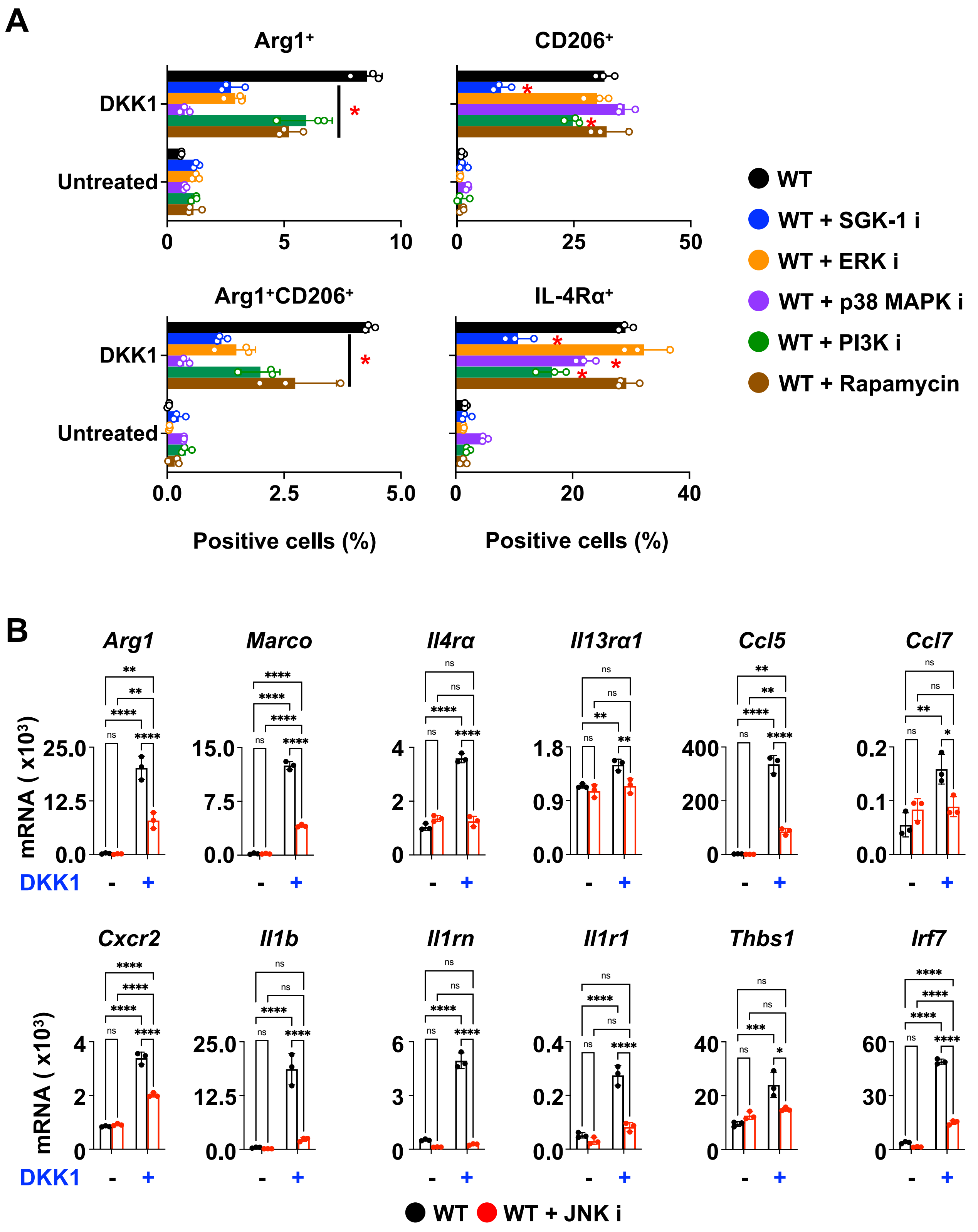

Supplementary Figure 4

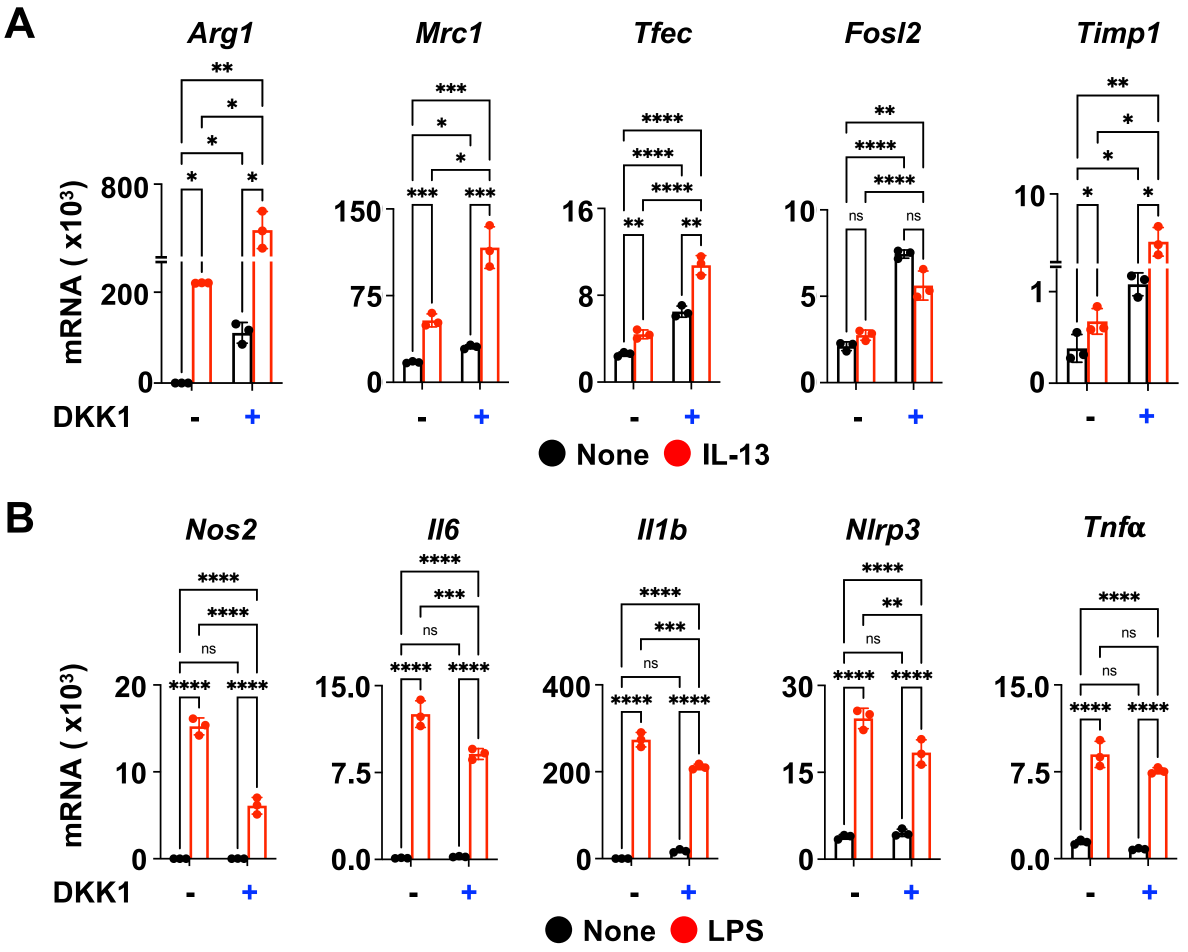

Supplementary Figure 4 (continued)

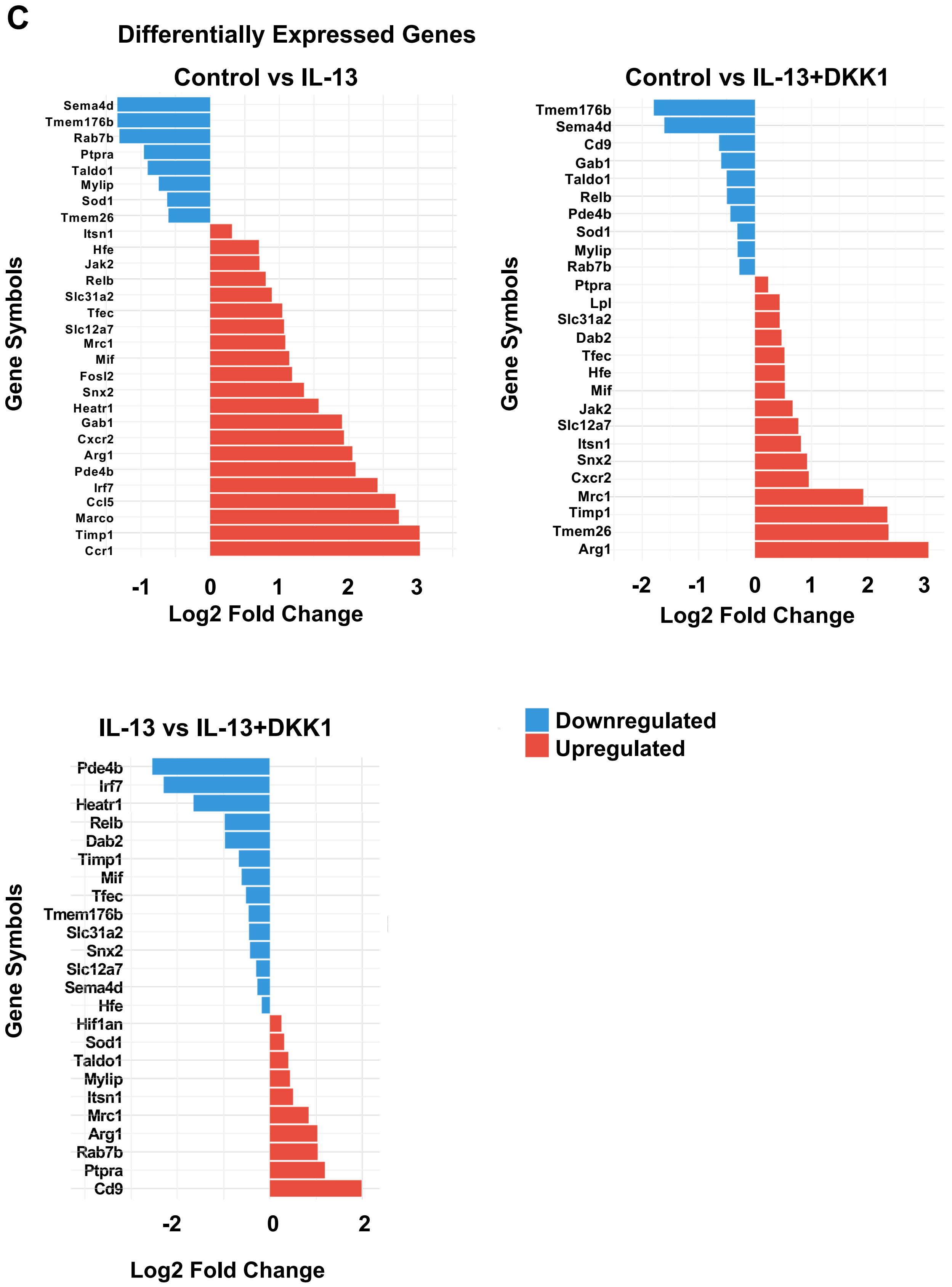

Supplementary Figure 4 (continued)

D

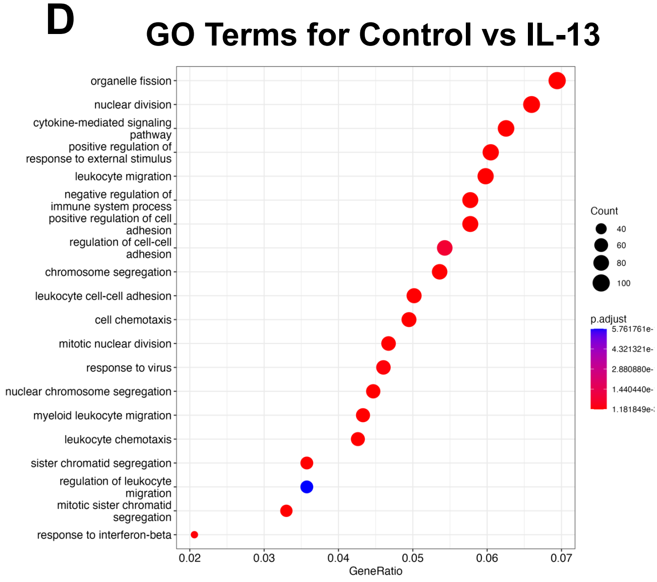

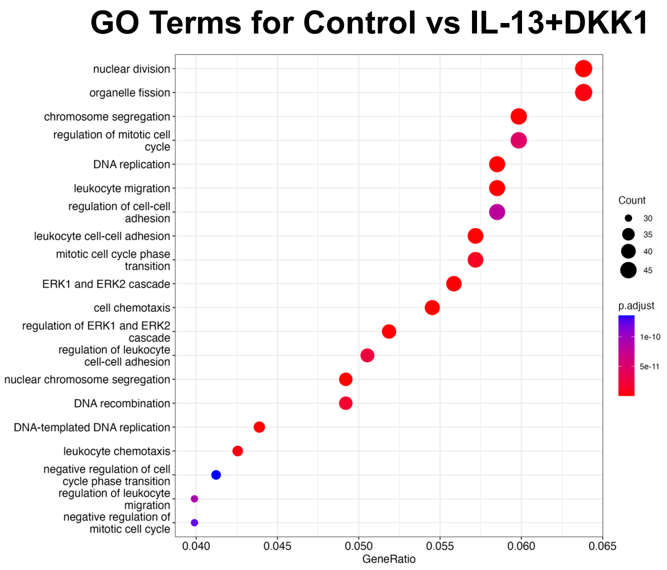

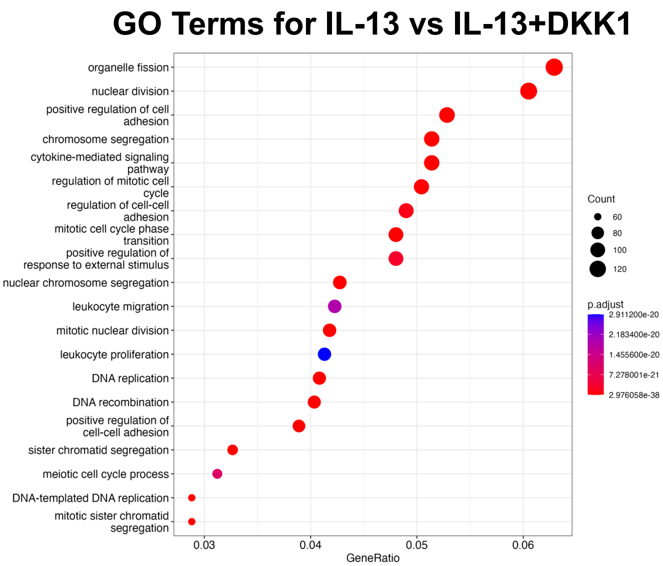

Supplementary Figure 4 (continued)

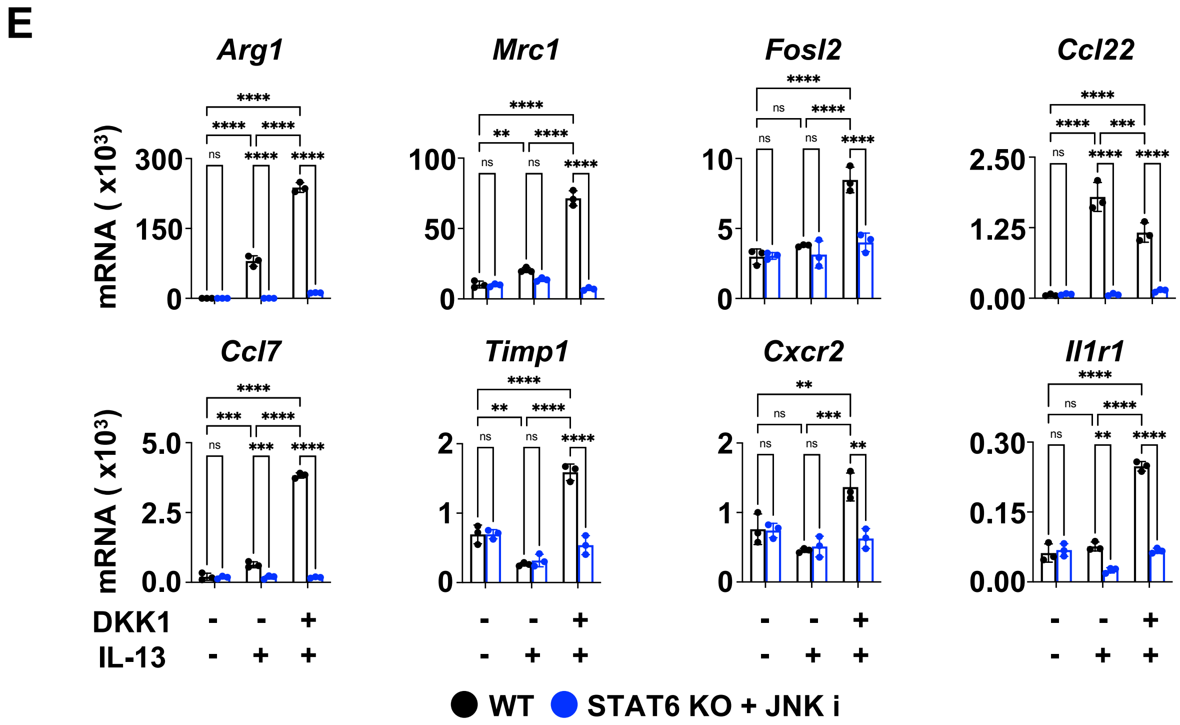

Supplementary Figure 5

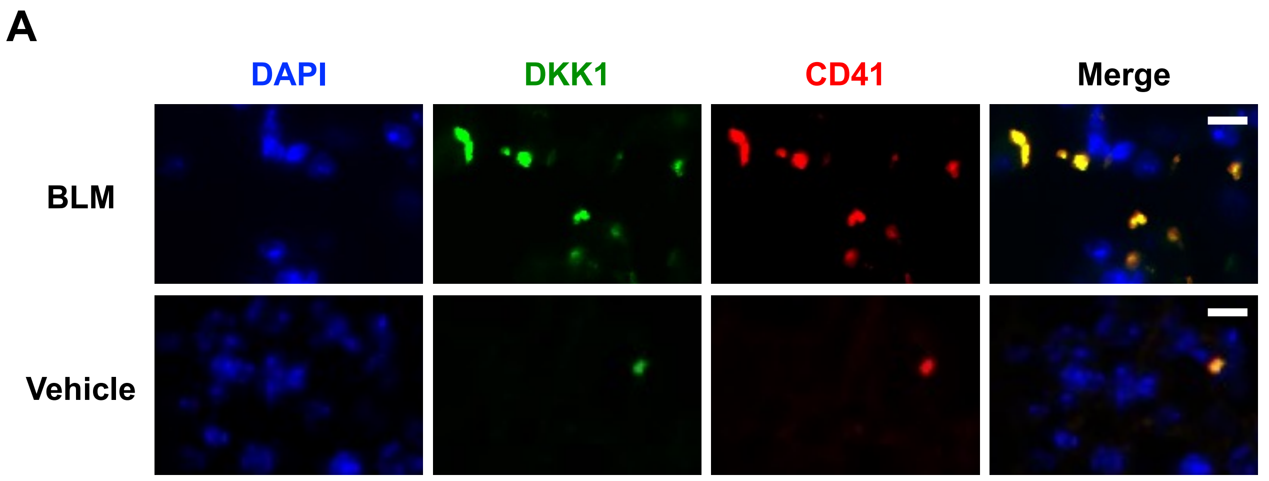

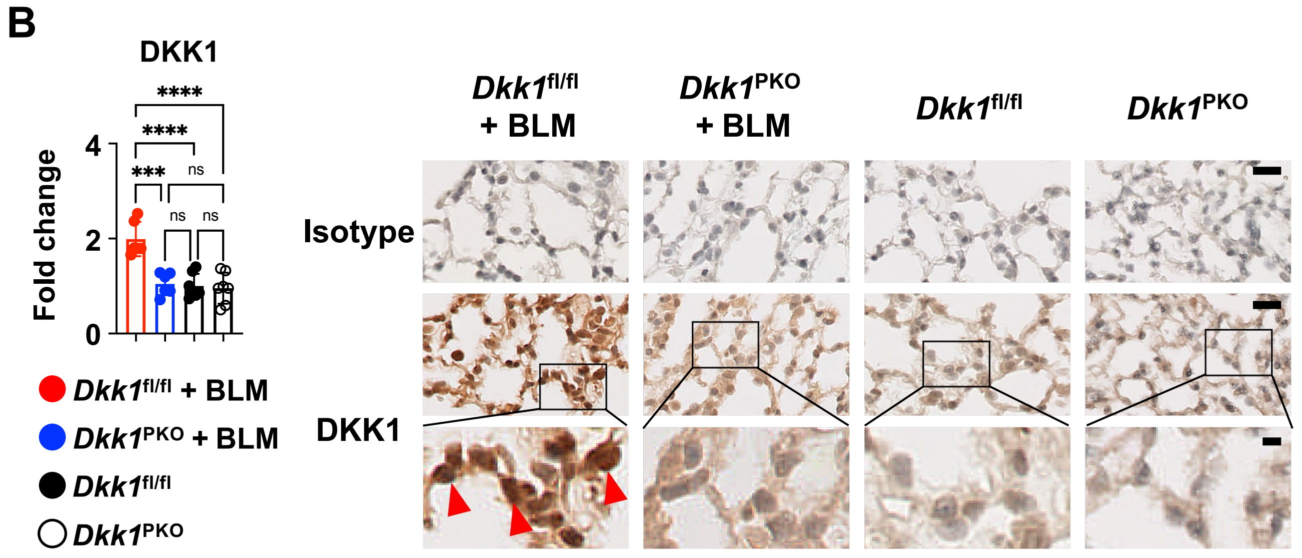

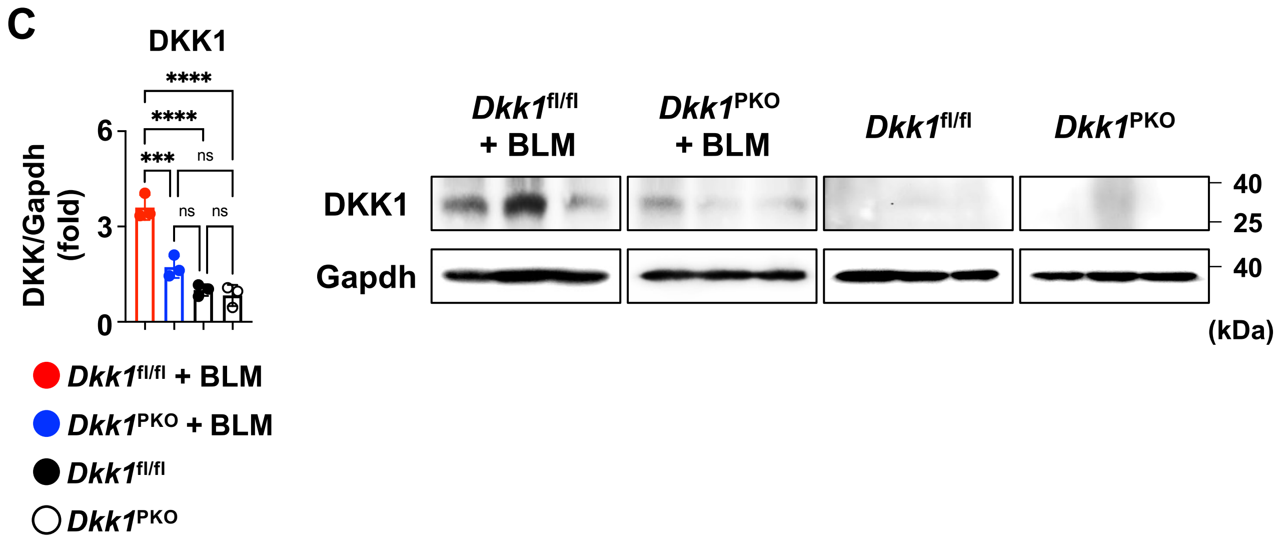

Supplementary Figure 6

A

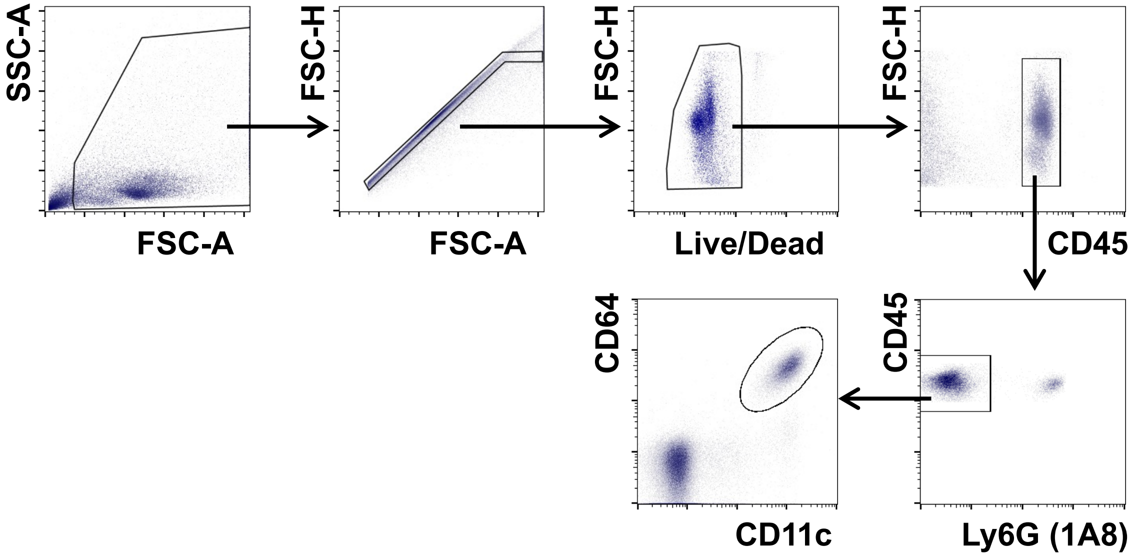

B

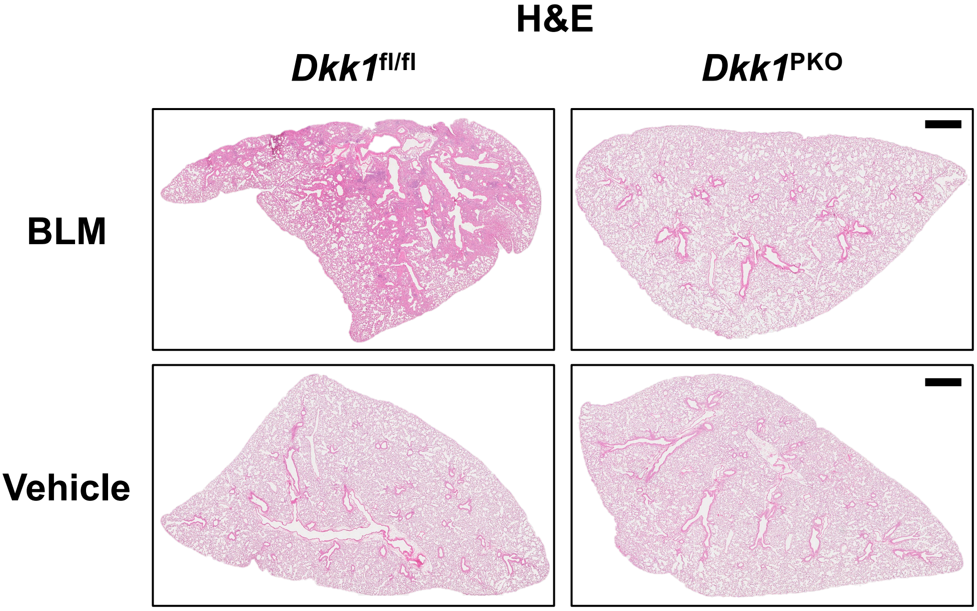

Supplementary Figure 6 (continued)

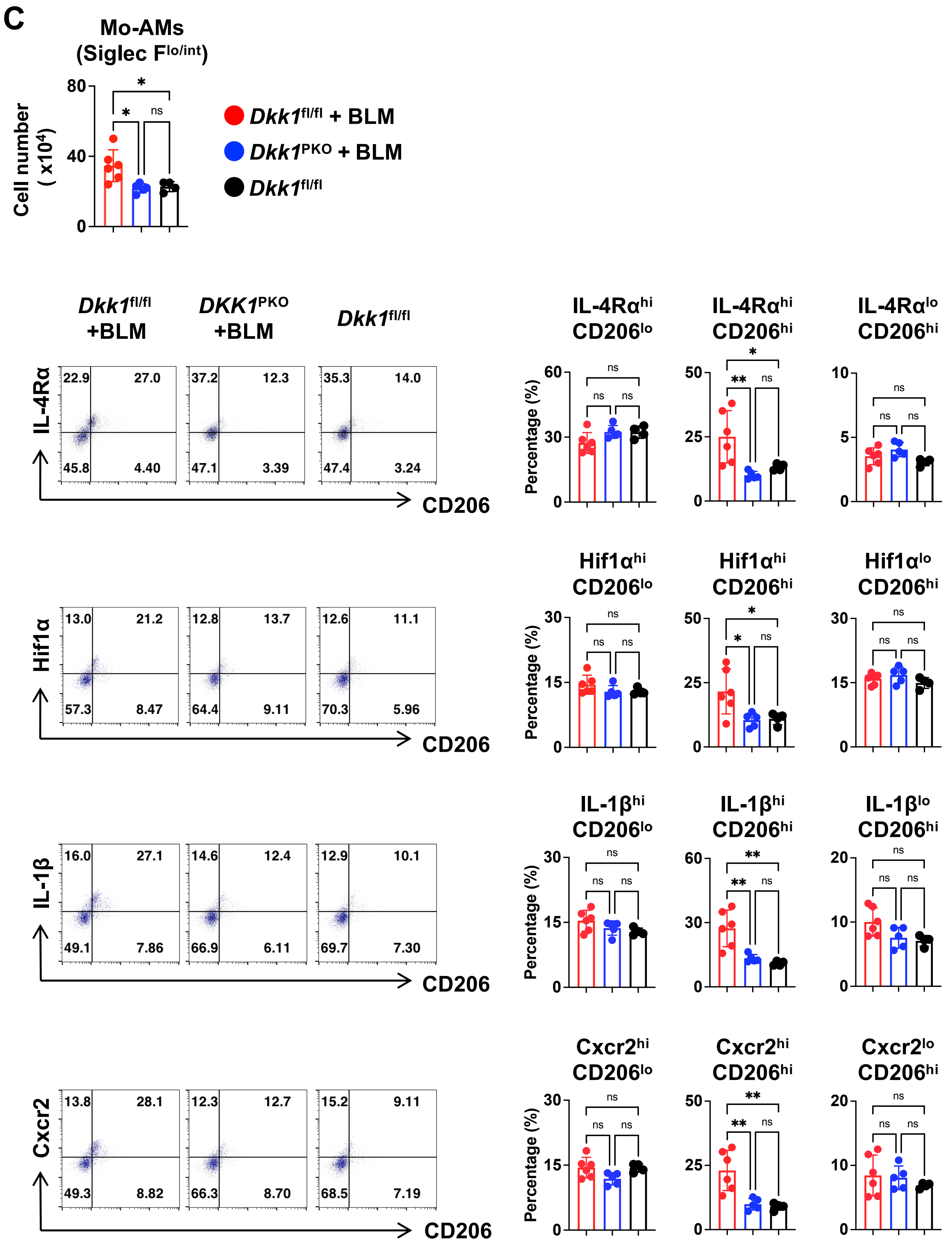

Supplementary Figure 6 (continued)

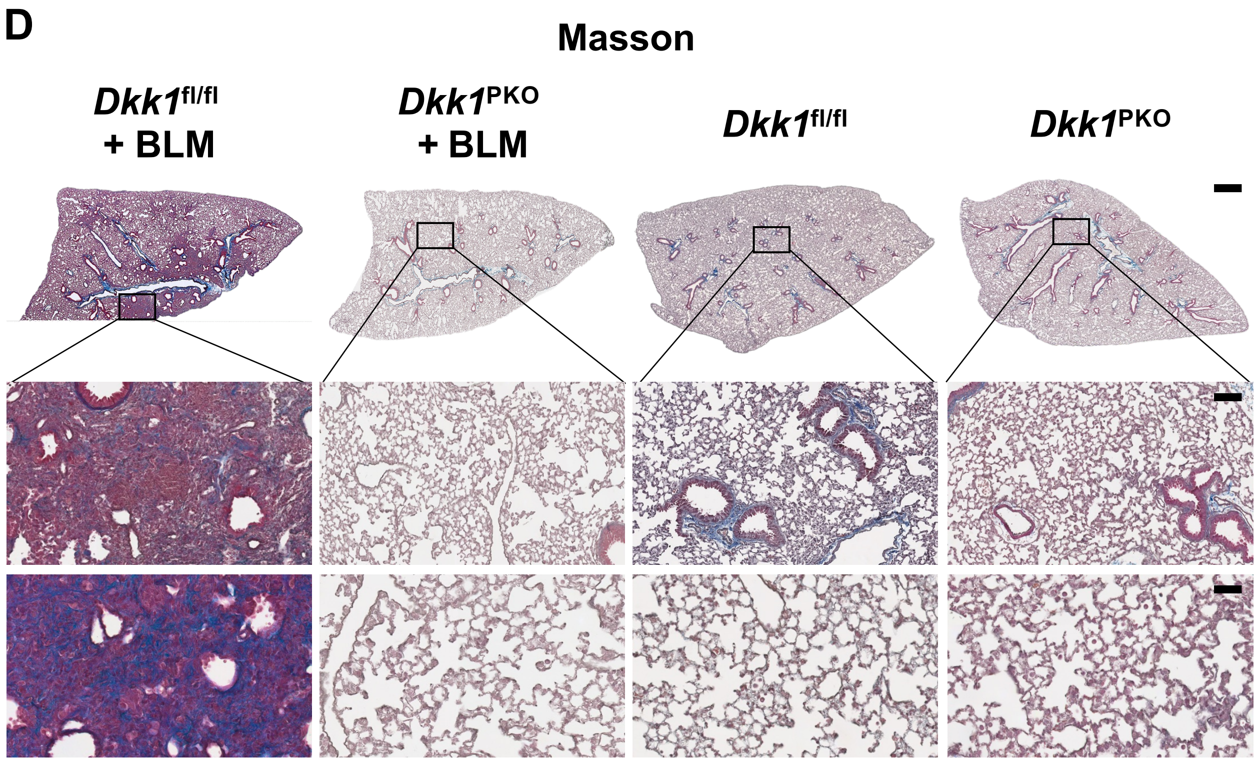

Supplement: Supplementary file 5 [file DataSheet_5.pdf]
