# Supplementary material for: Thrombocyte-derived Dickkopf1 promotes macrophage polarization in the Bleomycin-induced lung injury model
Source: Front Immunol. 2023 Dec 15;14:1247330. doi: 10.3389/fimmu.2023.1247330 (PMC10757334; doi:10.3389/fimmu.2023.1247330)
Supplement: Supplementary file 6 [file DataSheet_6.docx]

Supplementary Material

Thrombocyte-derived Dickkopf1 induces wound-healing macrophage polarization

Eun-Ah Sung^1,2†^, Min Hee Park^1,2†^, SuJeong Song^1,2^, Hasan Alanya^3^, Octavian Henegariu^3^, Jinze Liu^5^, E Zeynep Erson-Omay^3^, Patricia J Sime^4^, and Wook-Jin Chae^1, 2, 6*^

*** Correspondence:** Wook-Jin Chae, [wook-jin.chae@vcuhealth.org](mailto:wook-jin.chae@vcuhealth.org)

# Supplementary Figures and Tables

## Supplementary Figures

**Supplementary Figure 1.**

(**A**) On day 7 after BMDM differentiation, CD45^+^CD11b^+^F4/80^+^ cell populations were measured by flow cytometry. (**B**-**C**) BMDMs were treated with DKK1 for 24 hours prior to bulk RNA sequencing (RNAseq). DEG analysis between untreated and DKK1-treated groups is visualized in the form of a bar plot (**B**). Functional enrichment analysis was conducted to identify significantly up- and downregulated pathways in DKK1-treated group compared with untreated group (**C**). (**D**) BMDMs were stimulated with DKK1 for 24 hours prior to qPCR. (**E**) BMDMs were treated with DKK1 for 48 hours prior to flow cytometry analysis. IL-4Rα expressions in Arg1^+^CD206^-^ , Arg1^+^CD206^+^, and Arg1^-^CD206^+^ populations were quantified by flow cytometry. A representative of at least two independent experiments is shown. Student’s t-test was performed for (**D, E**). ****p < 0.0001, ***p < 0.001, **p < 0.005.

**Supplementary Figure 2.**

BMDMs from C57Bl/6J mice (WT) or STAT6 knockout mice (STAT6 KO) were treated with IL-13 for 24 hours prior to qPCR. A representative of at least two independent experiments is shown. A two-way ANOVA analysis with Bonferroni’s post hoc test was performed. ****p < 0.0001, ***p < 0.001, **p < 0.005, ns, not significant.

**Supplementary Figure 3.**

(**A**) BMDMs from C57Bl/6J mice (WT) were stimulated with JNK inhibitor with or without DKK1 for 24 hours prior to qPCR. (**B**) BMDMs were stimulated with the indicated inhibitors with or without DKK1 for 48 hours prior to flow cytometry analysis. Significant results (p-value < 0.05) are indicated as red asterisks. A representative of at least two independent experiments is shown. A two-way ANOVA analysis with Bonferroni’s post hoc test was performed for (**A**, **B**). ****p < 0.0001, ***p < 0.001, **p < 0.005, *p < 0.05, ns, not significant.

**Supplementary Figure 4.**

(**A**-**B**) BMDMs were treated with or without DKK1 and IL-13 (**A**) or LPS (**B**) for 24 hours prior to qPCR. (**C**-**D**) BMDMs were treated with IL-13 with or without DKK1 for 24 hours prior to bulk RNA seq. DEG analysis for three distinct comparisons is visualized in the form of a bar plot (**C**). Functional enrichment analysis was conducted to identify significantly up- and downregulated pathways in each group for three distinct comparisons (**D**). (**E**) WT or STAT6-deficient BMDMs were treated with IL-13 and/or DKK1 either with or without JNK inhibitor for 24 hours prior to qPCR. A representative of at least two independent experiments is shown. A two-way ANOVA analysis with Bonferroni's post hoc test was performed for (**A**, **B**, **E**). ****p < 0.0001, ***p < 0.001, **p < 0.005, *p < 0.05, ns, not significant.

**Supplementary Figure 5.**

(**A**) The lungs from C57Bl/6J mice were harvested on day 14 after the BLM challenge. Co-localization of CD41 and DKK1 was determined by immunofluorescence staining. Bar = 10 μm. (**B**) Dkk1^PKO^ mice and their Dkk1^fl/fl^ littermate controls were challenged with BLM. On day 14 after the challenge, the lungs were harvested. Relative protein levels of DKK1 were analyzed by IHC and Image J (left). Representative images of IHC are shown (right). The boxed areas are shown in higher magnification below. DKK1-positive cells are indicated by arrowheads. Bar = 20 μm (top and middle panels); 5 μm (bottom panels). Dkk1^fl/fl^ + BLM (n = 6); Dkk1^PKO^ + BLM (n = 6); Dkk1^fl/fl^ (n= 7); Dkk1^PKO^ (n = 7). (**C**) The lungs from Dkk1^PKO^ mice and their Dkk1^fl/fl^ littermate controls were harvested on day 14 after the BLM challenge. The ratio of DKK1 to Gapdh was quantified by Western blot and Image J. n= 3 per group. A one-way ANOVA analysis with Bonferroni's post hoc test was performed for (**B, C**). ****p < 0.0001, ***p < 0.001, ns, not significant.

**Supplementary Figure 6.**

(**A**) Flow cytometry gating strategy used for identification of macrophages (CD45^+^Ly6G (Gr-1, clone 1A8)^-^CD11c^+^CD64^+^ cells) in mouse lung homogenates is shown.

(**B**-**D**) Dkk1^PKO^ mice and their Dkk1^fl/fl^ littermate controls were challenged with BLM. On day 14 after the challenge, the lungs were harvested. (**B**) Representative images of H&E staining are shown. Bar = 800 μm. (**C)** Total Siglec F^lo^ Mo-AMs and protein expression levels of IL-4Rα, Hif1α, IL-1β, and Cxcr2 in CD206^+^ Siglec F^lo^ Mo-AMs were quantified by flow cytometry. Dkk1^fl/fl^ + BLM (n = 6); Dkk1^PKO^ + BLM (n = 5); Dkk1^fl/fl^ (n= 4). (**D**) Representative images of Masson staining are shown. Bar = 800 μm (top), 10 μm (middle), 20 μm (bottom). A one-way ANOVA analysis with Bonferroni's post hoc test was performed for (**C**). ****p < 0.0001, ***p < 0.001, **p < 0.005, *p < 0.05, ns, not significant.

## Supplementary Tables

**Supplementary Table 1.** **Antibodies for flow cytometry analysis used in this study**

| **Antibody** | **Manufacturer** | **Catalog No.** |
| --- | --- | --- |
| APC/Fire 750 anti-mouse CD45, clone 30-F11 | BioLegend | 103154 |
| APC anti-human/mouse Arginase 1, clone A1exF5 | eBioscience | 17-3697-82 |
| APC Mouse IgG1 kappa Isotype Ctrl Antibody, clone MOPC-21 | BD Biosciences | 550854 |
| Biotin anti-mouse CD206 (MMR), clone C068C2 | BioLegend | 141713 |
| Biotin anti-mouse Ly-6G, clone 1A8 | BioLegend | 127603 |
| eFluor 660 anti-mouse CD170 (Siglec F), clone 1RNM44N | eBioscience | 50-1702-80 |
| PE anti-mouse CD124(IL-4Ralpha), clone I015F8 | BioLegend | 144803 |
| PE IL-1 beta Monoclonal Antibody, clone CRM56 | eBioscience | 12-7018-41 |
| PE HIF-1 alpha Monoclonal Antibody, clone Mgc3 | eBioscience | 12-7528-82 |
| PE Mouse CXCR2/IL-8RB Antibody, clone 242216 | R&D Systems | FAB2164-P-025 |
| PE Mouse IgG1 kappa Isotype Ctrl Antibody, clone P3.6.2.8.1 | eBioscience | 12-4714-82 |
| PE Streptavidin | BioLegend | 405204 |
| PerCP/Cyanine5.5 anti-mouse CD64 (FcγRI), clone X54-5/7.1 | BioLegend | 139308 |
| PerCP/Cyanine5.5 Rat IgG2a kappa Isotype Ctrl Antibody, clone RTK2758 | BioLegend | 400531 |
| PE/Cyanine7 anti-mouse CD11c, clone N418 | BioLegend | 117317 |
| PE/Cyanine7 Mouse IgG1 kappa Isotype Ctrl Antibody, clone MOPC-21 | BioLegend | 400125 |
| Ultra-LEAF^TM^ Purified anti-mouse CD16/32 Antibody | BioLegend | 101330 |

**Supplementary Table 2.** **qPCR primers used in this study**

| **Gene name** | **Forward primer** | **Reverse primer** |
| --- | --- | --- |
| *Arg1* | TTAGAGATTATCGGAGCGCCTTTC | CCGTGGTCTCTCACGTCATACTCT |
| *Ass1* | TCGCAGAGCTCGTATACACAGGTT | ATGTACACTTGGCCCTTGAAGACA |
| *Ccl22* | GTTTGGATCAAGCCCTTTGT | CTGGACTGTGTGAGATTGGG |
| *Ccl5* | GTCGTGTTTGTCACTCGAAGGAAC | TGACAGGGAAGCTATACAGGGTCA |
| *Ccl7* | CCCCAAGAGGAATCTCAAGAGCTA | CTATAGCCTCCTCGACCCACTTCT |
| *Cxcr2* | GAACCAAGCTGATCAAGGAGACCT | TGGCCAATAAAGGCATAGATGATG |
| *Fizz1* | AGACTATGAACAGATGGGCCTCCT | GGTCCAGTCAACGAGTAAGCACAG |
| *Fosl2* | TATCCACGCTCACATCCCTACAGT | GACAGCTGCTCATCTCTCCTTCTG |
| *Hif1α* | ATTTTTGGACACTGGTGGCT | ATGCAATGGTGAAATGCTGA |
| *Il6* | TAGTCCTTCCTACCCCAATTTCCA | ATGAATTGGATGGTCTTGGTCCTT |
| *Il13rα1* | AAAGTGCATCTCACCCCCTG | CTCCAGGCTGCTGTACCAAT |
| *Il1b* | GATCCCAAGCAATACCCAAAGAAG | CTCTGCTTGTGAGGTGCTGATGTA |
| *Il1r1* | TGGAAGTCTTGTGTGCCCTTATGT | TTTACTCCGAAGAAGCTCACGTTG |
| *Il1rn* | AAGACAAGCGCTTTACCTTCATCC | GCCTCTAGTGTTGTGCAGAGGAAC |
| *Il4rα* | CTGGGACAGTGACCTGGACTGT | ACTCTCCCTTTGCAGAACTCAGG |
| *Irf7* | CCACGGAAAATAGGGAAGAAGTGA | ATCACTAGAAAGCAGAGGGCTTGG |
| *Marco* | CTTAGCAGCTATGGAGGTGGCTCT | ACACCCGCATCTTCATTATGTACG |
| *Mrc1* | GGAGTGATGGAACCCCAGTG | ACCCTCCGGTACTACAGCAT |
| *Nlrp3* | GTGGACTGCGAGAGATTCTACAGC | GATCTGAACAACCTCCTGGTCCTT |
| *Nos2* | GCTACCACATTGAAGAAGCTGGTG | CATAGGAAAAGACTGCACCGAAGA |
| *Tbp* | GAATAAGAGAGCCACGGACAACTG | AAGCCCAACTTCTGCACAACTCTA |
| *Tfec* | GACCATTCTAAAGGCATCAGTGGA | TAGTTCCTGAATCCGGAGCCTAAG |
| *Thbs1* | TGGCCTCTCCTGTGATGAACTATC | CTTCAGCTCACTGACCAGCTCTCT |
| *Timp1* | ATGCTAAAAGGATTCAAGGCTGTG | ATGAGAAACTCTTCACTGCGGTTC |
| *Tnfα* | CTGGGACAGTGACCTGGACTGT | ACTCTCCCTTTGCAGAACTCAGG |
